# Racial and Ethnic Disparities in Occupational Health

**DOI:** 10.1001/jamahealthforum.2025.3495

**Published:** 2025-09-26

**Authors:** Michael Dworsky, Leslie I. Boden, Elizabeth C. Chase, Yvette C. Cozier, Sarah Edgington, Marc N. Elliott, Seth A. Seabury

**Affiliations:** 1 RAND, Santa Monica, California; 2 Department of Environmental Health, Boston University School of Public Health, Boston, Massachusetts; 3 Department of Epidemiology, Boston University School of Public Health, Boston, Massachusetts; 4 Department of Pharmaceutical and Health Economics, USC Mann School, University of Southern California, Los Angeles

## Abstract

**Importance:**

Occupational health disparities affect the safety and well-being of workers across different racial and ethnic groups. Understanding these disparities is crucial for developing targeted interventions, but evidence on occupational injury rates by race and ethnicity has been scarce due to data limitations.

**Objective:**

To determine the incidence of lost-time workplace injuries among major racial and ethnic groups in California and to assess the contributions of occupational concentration and within-occupation disparities to these differences.

**Design, Setting, and Participants:**

A cross-sectional study analyzing 15 years (2005-2019) of California workers’ compensation data was carried out. The study examined injury rates across 4 racial and ethnic groups: Asian/Pacific Islander (non-Hispanic), Black (non-Hispanic), Hispanic, and White (non-Hispanic). California residents employed in the private sector or state and local government, encompassing diverse industries and occupations, between 2005 and 2019 were included. Analysis was conducted between May 2024 and May 2025.

**Intervention:**

The study analyzed existing workers’ compensation data without specific interventions.

**Main Outcomes and Measures:**

The primary outcome was the incidence of lost-time workplace injuries, with comparisons made between racial and ethnic groups. The analysis focused on the role of occupational concentration and within-occupation disparities in explaining injury rates.

**Results:**

The analysis included 2.6 million lost-time injuries among California workers (mean [SD] age, 42 [11]; 63% male). The overall injury rate was 1.32 lost-time injuries per 100 full-time equivalent workers (FTE). Lost-time injury rates were higher for Black (1.74 cases per 100 FTE) and Hispanic workers (1.90 cases per 100 FTE) than for White workers (1.00 cases per 100 FTE), whereas rates were lower for Asian/Pacific Islander workers (0.63 cases per 100 FTE). Black and Hispanic workers experienced injury rates that were 74% and 90% higher, respectively, compared with White workers, whereas Asian/Pacific Islander workers had injury rates that were 37% lower. Occupational concentration accounted for 53% of the disparity between Black and White workers and 71% of the disparity between Hispanic and White workers. Notably, 56% of the excess risk of injuries for Black women was attributed to within-occupation disparities.

**Conclusions and Relevance:**

Disparities in workplace safety are a significant contributor to racial and ethnic health disparities. Addressing both occupational concentration and within-occupation disparities is essential for improving workplace safety and reducing health inequities among workers.

## Key Points

**Question:**

How do occupational injury rates vary with race and ethnicity, and how important is occupational concentration (differences in the occupation distribution across groups) compared to within-occupation disparities in injury risk?

**Findings:**

In this cross-sectional analysis of California workers’ compensation claims from 2005 to 2019, Black (non-Hispanic) and Hispanic workers experienced lost-time injury rates that were 74% and 90% higher, respectively, compared with White (non-Hispanic) workers. Occupational concentration accounted for 53% of the disparity between Black and White workers and 71% of the disparity between Hispanic and White workers.

**Meaning:**

Occupational concentration contributes to occupational health disparities, but within-occupation disparities in risk are also important for public health.

## Teaser

This cross-sectional study analyzed the incidence of lost-time workplace injuries among major racial and ethnic groups in California and assessed the contributions of occupational concentration and within-occupation disparities to these differences.

## Introduction

In 2021 and 2022, employers reported 2.2 million nonfatal occupational injuries and illnesses involving at least 1 day lost from work. (We used “injuries” to refer to both injuries and illnesses).^1^ Occupational injuries can lead to disruption of family life,^2,3^ lost earnings,^4,5,6^ long-term disability,^7^ health problems,^8^ and excess mortality.^9,10^ The total burden of nonfatal occupational injuries in the US was estimated to be $198 billion in 2007, or $291 billion in 2023 dollars.^11^

Less is known about how injury risk and the resulting burden vary across racial and ethnic groups. Studies measuring racial and ethnic disparities in nonfatal injury rates or quantifying the role of occupational concentration have reached differing conclusions about the presence and magnitude of racial and ethnic disparities: some found no differences,^12^ whereas others found substantial disparities between Black and Hispanic workers and White workers.^13,14,15,16^

Occupational concentration of Black, Hispanic, or Asian/Pacific Islander workers into more dangerous jobs could result in higher injury rates (we use “occupational concentration” to refer to differences in the distribution of occupations between groups without taking a stand on whether these differences are normatively undesirable). However, differences in job duties, hazard communication, training, or access to protective equipment could also result in differences in risk among workers in the same occupation. Although these concerns have been noted for more than 50 years,^17^ evidence remains scarce on the magnitude and mechanisms of occupational health disparities. Of the studies that found racial and ethnic disparities in injury rates, 2 found that adjusting for occupation eliminated disparities,^13,14^ whereas 1 did not.^18^ Studies varied in data sources, case definitions, and the level of detail of occupational classification.^14,16^

Research on racial and ethnic differences in workplace injury risk has been hampered by limited data that simultaneously capture exposure (hours worked), injury occurrence, and race and ethnicity. Federal statistics on workplace safety are based on employer-recorded injuries, but race and ethnicity information is missing in nearly half of cases,^19^ making these data unsuitable for directly measuring racial and ethnic disparities.

We provide new evidence on occupational health disparities using 15 years (2005-2019) of California workers’ compensation data. Focusing on nonfatal injuries that result in lost workdays, we examined the incidence of workplace injury across 4 mutually exclusive major racial and ethnic groups: non-Hispanic Asian/Pacific Islander (hereafter, Asian/Pacific Islander), non-Hispanic Black (hereafter, Black), Hispanic (of any race), and non-Hispanic White (hereafter, White).

## Methods

We analyzed workers’ compensation administrative claims data and public-use survey data to estimate the rate of nonfatal occupational injuries across racial and ethnic groups, age, sex, and occupation. We also quantified the contribution of occupational concentration to differences in risk across groups. The study was approved by RAND’s Human Subjects Protection Committee (study #2023-N0427), which waived written informed consent. We followed the Strengthening the Reporting of Observational Studies in Epidemiology (STROBE) reporting guidelines for cross-sectional studies.

### Data

The data for this study came from 2 sources: the California Workers Compensation Information System (WCIS) and the American Community Survey (ACS). The WCIS is a database maintained by the California Department of Industrial Relations containing information on all California workers’ compensation claims.^20^ The ACS is a continuously fielded and nationally representative household survey administered by the US Census Bureau.^21,22^

We used the WCIS to count workplace injuries occurring from 2005 to 2019 in strata of workers defined by race and ethnicity, sex, age, and occupation (eg, Black women Financial Clerks aged 18-29 years). We used ACS data from 2005 to 2019 to estimate the number of full-time equivalent (FTE) workers in the strata described; details are presented in Supplement 1 (1 FTE is equal to 2000 hours worked).

### Population of Interest and Case Definition

We focused on California residents aged 18 to 64 years covered by the California workers’ compensation system between 2005 and 2019. Our outcome was the rate per 100 FTE of nonfatal lost-time injuries: those involving benefit payments or a settlement for wage-replacement benefits based on temporary or permanent disability.

### Missing Data

The WCIS, like other workers’ compensation claims data, does not contain direct measures of workers’ race and ethnicity or Standard Occupational Classification (SOC) codes for their occupations. In addition, some variables included in the WCIS data had missingness rates between 0.03% and 14.9% (eTable 1 in Supplement 1).

We addressed these missing data using multiple imputation.^23^ To impute race and ethnicity, we used the modified Bayesian Improved First Name-Surname Geocoding (mBIFSG) algorithm, which uses name and residential address to estimate the probability that people belong to each of 6 mutually exclusive race and ethnicity categories: American Indian/Alaska Native non-Hispanic, Asian/Pacific Islander non-Hispanic, Black non-Hispanic, Hispanic, White non-Hispanic, and multiracial non-Hispanic.^24,25,26,27^ We used mBIFSG to generate probabilities of belonging to each race and ethnicity group for each worker. Validation in a general population shows that mBIFSG has very high accuracy for the 4 racial and ethnic groups we examined in this study.^28^ A Washington State study^16^ validated an earlier version of the algorithm (BISG) against a sample of self-reports in workers’ compensation data and found good performance for the 4 largest racial and ethnic groups that we focused on.

Independently of the imputation of race and ethnicity, we used the NIOSH Industry and Occupation Computerized Coding System (NIOCCS) to probabilistically assign SOC codes from the industry code and occupational text description on the claim.^29^ We used NIOCCS to generate probabilities of belonging to each of the 95 minor SOC occupations (using 3-digit SOC codes).

We then generated multiple imputations of occupation and race and ethnicity by repeatedly sampling from multinomial distributions defined by the probabilities estimated by NIOCCS and mBIFSG. Imputation was done separately for race and ethnicity and occupation. We imputed other missing data using Generalized Efficient Regression-Based Imputation with Latent Processes (GERBIL).^30^ In each imputed dataset, we counted the number of occupational injuries occurring in each stratification cell grouped by age (ages 18-29, 30-49, 50-64 years), sex (male/female), race and ethnicity, and occupation. We generated 15 imputations for all estimates, pooling estimates from each imputed dataset and obtaining variance estimates using Rubin rules. Details are available in Supplement 1.

### Statistical Analysis

Using the number of injuries as the outcome and the ACS-based estimate of the number of FTE workers as the exposure (offset) term, we used Poisson regression to estimate the association between race and ethnicity and occupational injury risk. We first fit an unadjusted model in which race and ethnicity was the only predictor. We then fit a model that also included sex, age category, and sex interacted with age category. Finally, we estimated a model that added an indicator variable for each occupation. These models were estimated by quasi-maximum likelihood using Huber-White heteroskedasticity-robust standard errors to adjust for heteroskedasticity.

We used these estimates to calculate adjusted occupational injury risk differences, comparing Black, Hispanic, and Asian/Pacific Islander workers to White workers. We calculated predicted rates and risk differences using the demographic and occupation distribution of White workers. The adjusted risk difference can be interpreted as the risk difference that would result if the demographic and occupation distributions of, for instance, Black workers were identical to the distributions for White workers.

To assess how much of the disparity in injury rates was driven by occupational concentration, we estimated risk differences with and without adjustment for occupation. If adjusting for occupation eliminated the risk differences, we could conclude that disparities were due entirely to occupational concentration. If adjusting for occupation reduced, but did not eliminate the disparity, we could interpret the percentage change in the risk difference as the proportion of the disparity attributable to occupational concentration. We compared estimated incidence rate ratios across models with and without adjustment for occupation to test whether the disparity attributable to occupational concentration was statistically significantly different from zero.

Finally, we conducted subgroup analyses stratified by sex to examine the possibility that the magnitude of disparities and the role of occupational concentration differed by sex. We also estimated models stratified by occupation to explore how the racial and ethnic disparity within occupations varied after adjusting for demographic differences. Statistical significance was evaluated using 2-sided tests with α = .05. Analyses were carried out using Stata (version 18.0, StataCorp) and R statistical software, (version 4.4.1, R Foundation for Statistical Computing).

## Results

The data included 2.6 million lost-time injuries over the 2005 to 2019 period (Table). Among injured workers, 37% were female and the mean (SD) age at injury was 41.5 (11.3) years. Age and sex differences between injured workers in different racial and ethnic groups were apparent: women accounted for a larger share of injured workers among Asian/Pacific Islander and Black workers, whereas the mean (SD) age at injury for Hispanic workers was 38.7 (11.3) years, nearly 3 years younger than the overall average. Hispanic workers (53%) made up more than half of those injured in California from 2005 to 2019. The population at risk contained 195 million FTE workers over the 15-year study period. As the Table shows, we estimate that the population of workers with lost-time injuries was 53.0% Hispanic, 30.9% White, 7.1% Asian/Pacific Islander, 6.7% Black, 2.0% Multiracial, and 0.4% American Indian/Alaska Native.

**Table.** Lost-Time Workers’ Compensation Claims and Full-Time Equivalent (FTE) Employment in California by Race, Ethnicity, and Sex, 2005 to 2019^a^

Variable	American Indian/Alaska Native non-Hispanic	Asian/Pacific Islander non-Hispanic	Black non-Hispanic	Hispanic	Multiracial non-Hispanic	White non-Hispanic	Total
**All workers**							
Female, %	41	45	49	33	43	37	37
Age at injury, mean (SD), y	42 (12)	42 (11)	42 (11)	39 (11)	42 (11)	42 (11)	42 (11)
Lost-time injuries, 2005-2019, No. (%)	9625 (0.4)	183 593 (7.1)	171 231 (6.7)	1 363 718 (53.0)	52 357 (2.0)	794 357 (30.9)	2 574 881 (100)
FTE, 2005-2019 (million FTE), No. (%)	611 326 (0.3)	29 284 392 (15.0)	9 849 858 (5.1)	71 700 286 (36.8)	3 945 926 (2.0)	79 529 213 (40.8)	194 921 001 (100)
Unadjusted injury rate per 100 FTE	1.57	0.63	1.74	1.90	1.33	1.00	1.32
**Men**							
Female, %	0	0	0	0	0	0	0
Age at injury, mean (SD), y	41 (12)	41 (11)	42 (11)	38 (11)	41 (11)	42 (11)	41 (11)
Lost-time injuries, 2005-2019, No. (%)	5310 (0.3)	95 434 (6.2)	80 651 (5.3)	847 718 (55.3)	27 568 (1.8)	476 817 (31.1)	1 533 498 (100)
No. of FTE, 2005-2019 (million FTE), No. (%)	314 703 (0.3)	15 350 114 (13.9)	4 824 261 (4.4)	43 304 750 (39.3)	2 049 923 (1.9)	44 271 047 (40.2)	110 114 798 (100)
Unadjusted injury rate per 100 FTE	1.69	0.62	1.67	1.96	1.34	1.08	1.39
**Women**							
Female, %	100	100	100	100	100	100	100
Age at injury, mean (SD), y	43 (11)	44 (11)	42 (11)	41 (11)	43 (11)	44 (11)	43 (11)
No. of lost-time injuries, 2005-2019, No. (%)	4315 (0.4)	88 159 (8.5)	90 580 (8.7)	516 000 (49.5)	24 790 (2.4)	317 540 (30.5)	1 041 383 (100)
No. of FTE, 2005-2019 (million FTE), No. (%)	296 623 (0.3)	13 934 278 (16.4)	5 025 597 (5.9)	28 395 536 (33.5)	1 896 003 (2.2)	35 258 166 (41.6)	84 806 203 (100)
Unadjusted injury rate per 100 FTE	1.45	0.63	1.80	1.82	1.31	0.90	1.23

^a^ Authors’ calculations, 2005 to 2019 California Workers Compensation Information System (WCIS) and American Community Survey (ACS). All estimates other than FTE (which were estimated using the ACS) are based on 15 multiply imputed datasets (Supplement 1).

The overall injury rate was 1.32 lost-time injuries per 100 FTE. Lost-time injury rates were far higher for Black (1.74 cases per 100 FTE) and Hispanic workers (1.90 cases per 100 FTE) than for White workers (1.00 cases per 100 FTE), whereas rates were lower for Asian/Pacific Islander workers (0.63 cases per 100 FTE).

**Figure 1.**
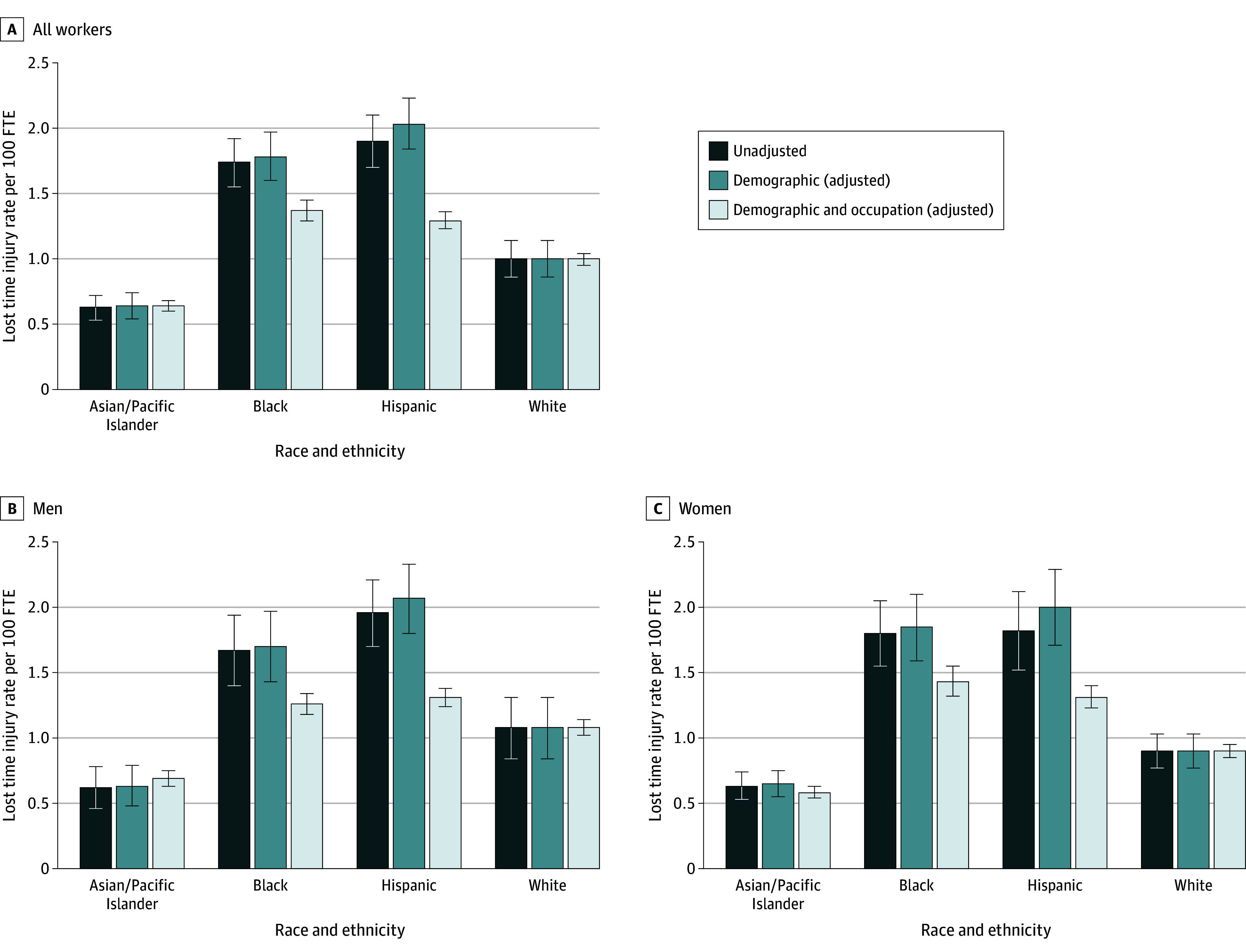
Unadjusted and Adjusted Lost-Time Injury Incidence Rates per 100 Full-Time Equivalent (FTE) and Contribution of Occupational Concentration to Demographic-Adjusted Disparity, by Race, Ethnicity, and Sex All groups other than Hispanic are non-Hispanic. Authors’ calculations, 2005 to 2019 California Workers Compensation Information System (WCIS) and American Community Survey (ACS). Bars indicate predicted incidence rates from Poisson regression models for lost-time injury counts. Error bars (whiskers) indicate 95% CIs based on heteroskedasticity-robust Huber-White standard errors. Estimates based on 15 multiply imputed datasets; see Supplement 1 for details. The number of FTE workers (in hundreds) is used as an exposure term (ie, ln[FTE/100] is included with the coefficient constrained to equal 1). All models included an intercept and indicators for race and ethnicity (White is the excluded category; American Indian/Alaska Native non-Hispanic and multiracial non-Hispanic were excluded from the sample). Demographic-adjusted models also included indicators for sex (excluded category was men), age (excluded category was ages 18-29 years), and sex-age interactions in models for all workers. Demographic-adjusted models stratified by sex included only age category indicators. Demographic and occupation-adjusted models also included indicators for 95 occupation categories (excluded category was top executives [SOC 11-1000]). Adjusted incidence rates are predicted injury rates per 100 FTE under the distribution of included covariates observed for White workers.

Figure 1 shows predicted incidence rates and risk differences estimated in our regression models. Full regression tables are in Supplement 1.

Adjusting for age and sex resulted in a slightly larger risk difference between Hispanic workers (adjusted risk difference of 1.04 cases per 100 FTE vs unadjusted risk difference of 0.90 cases per 100 FTE; *P* = .002) and White workers, but had little impact on the disparities estimated for Asian/Pacific Islander and Black workers. Adjusting for occupation substantially reduced, but did not eliminate, the disparities between Black and Hispanic injury rates and White injury rates.

For Black workers, adjusting for occupation reduced the demographic-adjusted risk difference by 53%, from 0.78 injuries per 100 FTE to 0.37 injuries per 100 FTE. For Hispanic workers, adjusting for occupation reduced the demographic-adjusted risk difference by 71%, from 1.04 injuries per 100 FTE to 0.30 injuries per 100 FTE. Changes in the underlying incidence rate ratio from adjusting for occupation were statistically significant (*P* = .001 for Black workers, *P* < .001 for Hispanic workers) for both groups. For Asian/Pacific Islander workers, in contrast, the risk difference was unaffected by adjustment for occupation (*P* = .95).

**Figure 2.**
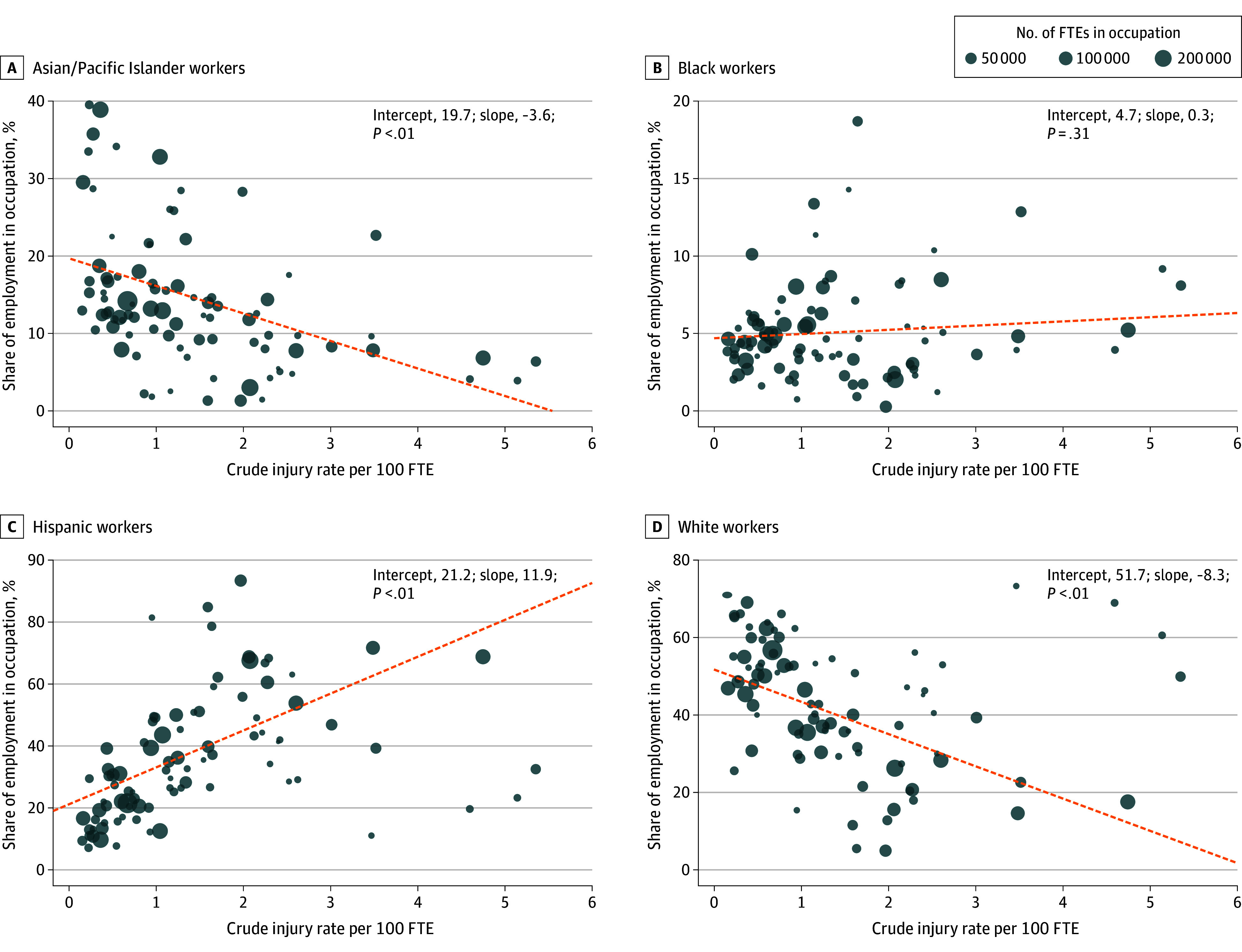
Occupational Employment Shares vs Overall Injury Rates, by Race and Ethnicity Authors’ calculations, 2005 to 2019 WCIS and ACS. Crude injury rate per 100 full-time equivalent (FTE) is calculated for all workers (ie, averaged over all racial and ethnic groups). The line of best fit reported in the graph is a weighted least squares regression of employment shares on the crude injury rate using FTE employment as weights. The *P *value is for statistical significance of the slope coefficient on the line of best fit. Estimates of incidence rates are based on 15 multiply imputed datasets; see Supplement 1 for details.

To illustrate how occupational concentration results in differential exposure to high-risk occupations, each panel of Figure 2 plots the proportion of workers by race and ethnicity in each of the 95 occupations we analyzed against the overall lost-time injury rate. For Asian/Pacific Islander and White workers, the employment share in an occupation declined with the injury rate. For Black workers, the employment share was not associated with the injury rate, whereas for Hispanic workers, the employment share rose sharply with the injury rate. These differences in the relationship between injury risk and employment shares help explain why adjusting for occupation does so much to reduce the risk differences.

### Estimated Racial and Ethnic Disparities by Sex

Figure 1 also shows adjusted rates stratified by sex. The direction of the unadjusted disparities and the effects of adjustment for age were similar for male and female workers, with higher injury rates for Black and Hispanic workers and lower injury rates for Asian/Pacific Islander workers compared with White workers. Similarly, age-adjusted disparities were larger than unadjusted disparities for Hispanic workers (*P* = .01 for men and *P* = .02 for women), but not meaningfully different from unadjusted disparities for Asian/Pacific Islander and Black workers.

When we adjusted for occupation, however, we found sex differences in the importance of occupational concentration relative to within-occupation disparities for Black and Hispanic workers.

For men, occupational concentration accounts for most of the demographic-adjusted disparity in both groups. Adjusting for occupation reduced the disparity for Black men by 70%, from 0.62 injuries to 0.19 injuries per 100 FTE, and adjusting for occupation reduced the disparity for Hispanic men by 77%, from 0.99 injuries to 0.23 injuries per 100 FTE.

For women, occupational concentration explains less of the demographic-adjusted disparity. For Hispanic women, occupational concentration still accounted for the majority (63%) of the age-adjusted disparity, reducing the disparity from 1.10 injuries to 0.41 injuries per 100 FTE.

For Black women, in contrast, adjusting for occupation explained only 44% of the age-adjusted disparity, reducing the disparity from 0.95 cases to 0.53 cases per 100 FTE. The within-occupation risk difference for Black women was nearly triple the within-occupation risk difference for Black men, whereas the within-occupation risk difference for Hispanic women was 77% higher than the within-occupation risk difference for Hispanic men.

### Within-Occupation Disparities

Although these results highlight occupational concentration as an explanation for the higher injury rates experienced by Black and Hispanic workers compared with White workers, within-occupation disparities for these groups remained substantial, most notably for Black women. We accordingly estimated within-occupation disparities (risk differences adjusted for demographics) for each of the 95 occupations examined in our study by stratifying our regression model on occupation.

**Figure 3.**
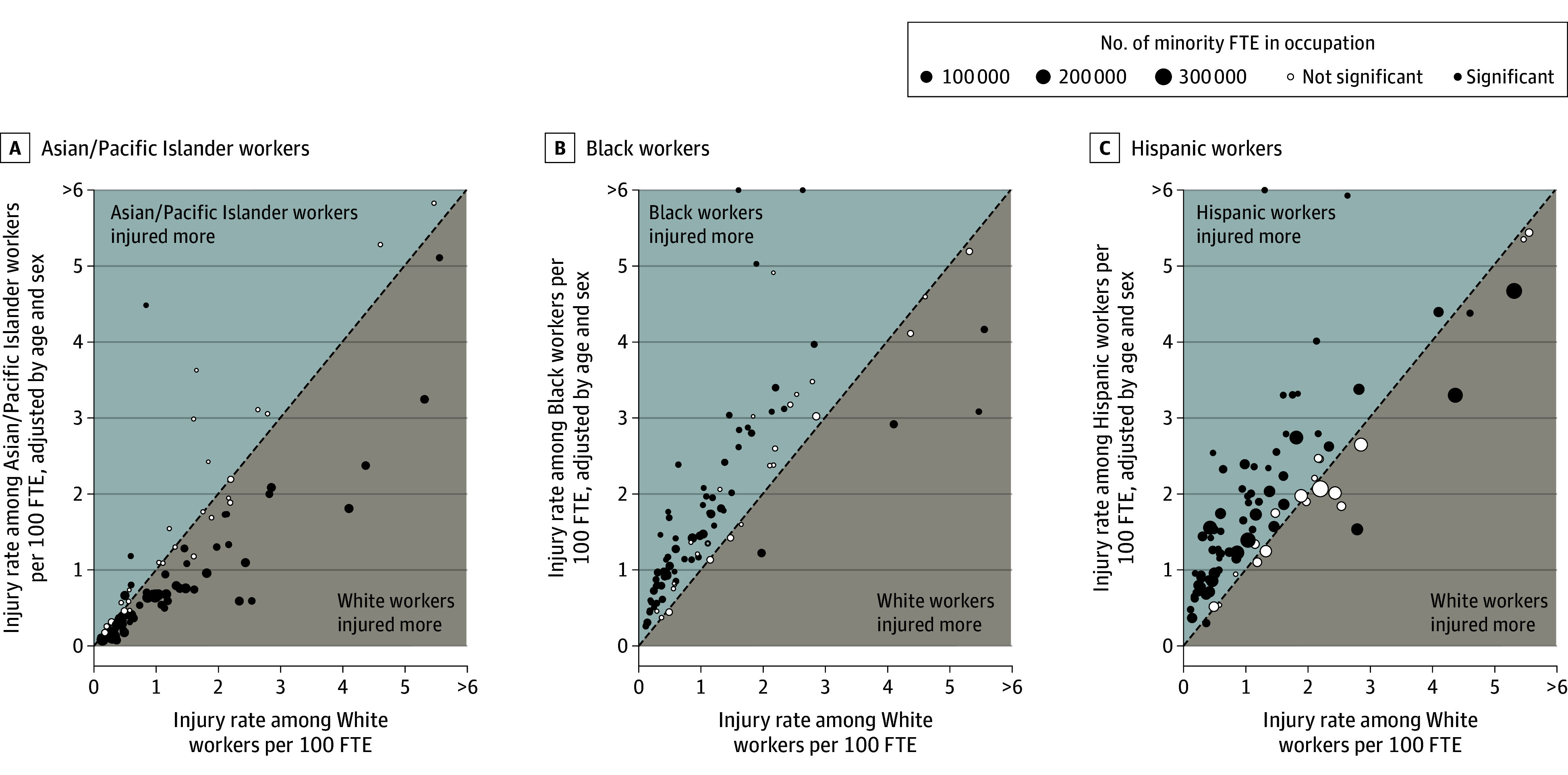
Within-Occupation Demographic-Adjusted Injury Rates vs White Injury Rates, by Race and Ethnicity and Occupation Authors’ calculations, 2005-2019 California Workers Compensation Information System (WCIS) and American Community Survey (ACS). The dashed line is the 45-degree line. Significant (or not significant) indicates statistical significance (or insignificance) at the 5% level of the difference between the adjusted rate for each group and the injury rate among White workers within each occupation. Estimates based on 15 multiply imputed datasets; see Supplement 1 for details. The injury rate for each group is adjusted for demographic (age and sex) differences from the White non-Hispanic population using a Poisson regression with the number of full-time equivalent (FTE) workers (in hundreds) included as an exposure term (ie, ln[FTE/100]) is included with the coefficient constrained to equal 1, an intercept, indicators for race/ethnicity categories (WNH is the excluded category; non-Hispanic American Indian/Alaska Native and multiracial non-Hispanic were excluded from the sample), and indicators for sex (excluded category was men), age (excluded category was ages 18-29 years), and sex-age interactions. All groups other than Hispanic are non-Hispanic. Models are stratified by 95 occupation categories.

Figure 3 presents the estimated within-occupation disparities, plotting the injury rate in each occupation adjusted for age and sex (ie, the incidence rate from the occupation-specific model predicted under the White age and sex distribution) against the rate observed for White workers.

For both Black and Hispanic workers, within-occupation risk differences were positive for most occupations examined. The demographic-adjusted injury rate among Black workers was statistically significantly greater than the rate among White workers in 61 of 95 occupations, whereas the injury rate among Black workers was significantly lower than the rate among White workers in only 5 of 95 occupations. Likewise, the demographic-adjusted injury rate among Hispanic workers was significantly greater than the rate among White workers in 69 of 94 occupations, whereas the injury rate among Hispanic workers was significantly lower than the rate among White workers in only 6 of 94 occupations (within-occupation disparities for Hispanic vs White workers in SOC 45-3000 [fishing and hunting workers] could not be estimated due to insufficient data).

We concluded that occupation-adjusted disparities between Black and Hispanic and White workers (Figure 1) were not driven by a small number of occupations or differentially driven by low- or high-risk occupations. Instead, within-occupation disparities were pervasive: even some lower-risk occupations have substantial within-occupation disparities that contribute to the overall disparities reported herein. Supplement 2 (the Incidence Disparities Supplement Workbook) consists of an Excel Workbook that reports unadjusted occupation-specific rates by race and ethnicity for all occupations with at least 1500 FTE and more than 10 injuries for each racial and ethnic group.

Supplement 2 also presents occupation-specific rates by race and ethnicity stratified by sex. These resemble the overall estimates: within-occupation disparities for men and women overwhelmingly have the same sign as the within-occupation effects reported in the Table, indicating that within-occupation disparities in injury rates were widespread and were not driven by a small set of occupations.

## Discussion

This cross-sectional study analyzed how lost-time injury rates varied across racial and ethnic groups in California from 2005 to 2019, finding that Black and Hispanic workers experienced workplace injuries at far higher rates than White workers. These disparities reflect both occupational concentration and elevated risk compared with workers within the same occupation. We also found previously unknown sex differences in the role of occupational concentration: occupational concentration explains a greater proportion of the overall disparity for men (70% for Black men and 76% for Hispanic men) than for women (44% for Black women and 63% for Hispanic women).

### Limitations

Underreporting of injuries is a limitation of all occupational injury research. As with the federal Survey of Occupational Injuries and Illnesses (SOII), which is the main source of federal statistics on occupational injury rates, many workplace injuries are not recorded by workers’ compensation.^31,32,33,34^ One study using 2007 to 2008 data from California estimated that workers’ compensation captures about 75% of lost-time injuries.^32^ This is comparable to estimates of the completeness of the SOII in California during our study period, suggesting that, although our data may be incomplete, they likely represent the most comprehensive available set of information on workplace injuries.^35^

We also note that, if anything, this limitation suggests our findings might understate the burden of workplace injuries to Asian/Pacific Islander, Black, or Hispanic workers. If, as suggested by past work, underreporting is associated with dimensions of social vulnerability (like English language proficiency, precarious work arrangements, or the threat of employer retaliation)^33^ that are more pronounced in the Black and Hispanic populations, our study may underestimate the true disparities in injury risk between these groups and the White population.

Another limitation is that we did not directly observe race and ethnicity and had to impute it based on other observable factors. This limitation is common to most large administrative databases, including health care claims, and the methods we used have been well validated in prior work. Nonetheless, it is possible that we may have incorrectly imputed race and ethnicity for some individuals in the claims data, possibly leading to biased estimates of the disparities. Future work should link data with self-reported race and ethnicity information for California workers’ compensation claims, enabling us to assess the validity of mBIFSG in this setting. Given that such data were unavailable, mBIFSG offers a validated technique that allows us to examine racial and ethnic disparities in occupational safety.

A related limitation is that we could not examine more disaggregated racial and ethnic groups than those estimated by mBIFSG. Data on more disaggregated Asian and Pacific Islander and Hispanic subpopulations^36^ could uncover policy-relevant heterogeneity in occupational exposures and within-occupation injury risks faced by these subgroups.

## Conclusion

We found substantial racial and ethnic disparities in nonfatal injury rates, and we also found that differences in occupations accounted for much of these disparities. Although past research in this area has been inconsistent, our results combined with some other recent studies would suggest these past inconsistencies are likely due to data limitations and/or a focus on small convenience samples. The closest analog to our study, a recent study^16^ in Washington State using similar methods, found nonfatal injury rates among Black workers were substantially higher than for Hispanic and White workers within broad occupation and industry categories and also found disparities between Hispanic and White workers for most of these categories. Our work builds on this and other studies by using far more detailed information about occupation and providing a more complete assessment of the extent to which disparities in workplace injury can be explained by occupational concentration.

The fact that risk differences persist even when comparing workers in the same jobs, particularly for Black and women workers, suggests that Black and Hispanic workers systematically face worse working conditions and riskier environments. Past work has shown that workplace injuries and exposure to adverse working conditions contribute to poor health and disability. Our findings suggest that the occupational health disparities identified herein could be an important contributing factor to overall health disparities. Efforts to alleviate health disparities should consider the role of working conditions, not just economic outcomes such as income, as a potentially important, modifiable factor.

## Notes

Supplement 1. eMethods eTable 1. Rates of Missing Data Before Multiple Imputation eTable 2. ACS Sample Construction eTable 3. ACS Sample Construction eTable 4. Denominator Summary Statistics and Marginal Probabilities eFigure 1. Racial/Ethnic Composition by Occupation: Total eFigure 2. Racial/Ethnic Composition by Occupation: Men eFigure 3. Racial/Ethnic Composition by Occupation: Women eTable 5. Lost-Time Injury Incidence Rates per 100 FTE with Adjustment for Demographics and Occupation, by Race/Ethnicity and Sex eFigure 4. Demographic-Adjusted Occupation-Specific Risk Differences between Black non-Hispanic Workers and White non-Hispanic Workers, Men and Women Pooled eFigure 5. Demographic-Adjusted Occupation-Specific Risk Differences between Hispanic Workers and White non-Hispanic Workers, Men and Women Pooled eFigure 6. Demographic-Adjusted Occupation-Specific Risk Differences between Asian/Pacific Islander non-Hispanic Workers and White non-Hispanic Workers, Men and Women Pooled eFigure 7. Demographic-Adjusted Occupation-Specific Risk Differences between Black non-Hispanic Workers and White non-Hispanic Workers, Men eFigure 8. Demographic-Adjusted Occupation-Specific Risk Differences between Hispanic Workers and White non-Hispanic Workers, Men eFigure 9. Demographic-Adjusted Occupation-Specific Risk Differences between Asian/Pacific Islander non-Hispanic Workers and White non-Hispanic Workers, Men eFigure 10. Demographic-Adjusted Occupation-Specific Risk Differences between Black non-Hispanic Workers and White non-Hispanic Workers, Women eFigure 11. Demographic-Adjusted Occupation-Specific Risk Differences between Hispanic Workers and White non-Hispanic Workers, Women eFigure 12. Demographic-Adjusted Occupation-Specific Risk Differences between Asian/Pacific Islander non-Hispanic Workers and White non-Hispanic Workers, Women eTable 6. Sensitivity Analysis: Lost-Time Injury Results Using Winsorized Rates eTable 7. Sensitivity Analysis: Excluding Small Cells eTable 8. Sensitivity Analysis: Results Under Alternative Injury Case Definitions eTable 9. Sensitivity Analysis: Complete Records Analysis eTable 10. Sensitivity Analysis: Excluding Predominantly Public-Sector Occupations and Temporary/Contract Employees

Supplement 2. Incidence Disparities Supplement Workbook

Supplement 3. Data Sharing Statement
