# Supplementary material for: Racial and Ethnic Disparities in Occupational Health
Source: JAMA Health Forum. 2025 Sep 26;6(9):e253495. doi: 10.1001/jamahealthforum.2025.3495 (PMC12475949; doi:10.1001/jamahealthforum.2025.3495)
Supplement: Supplement 1. — eMethods eTable 1. Rates of Missing Data Before Multiple Imputation eTable 2. ACS Sample Construction eTable 3. ACS Sample Construction eTable 4. Denominator Summary Statistics and Marginal Probabilities eFigure 1. Racial/Ethnic Composition by Occupation: Total eFigure 2. Racial/Ethnic Composition by Occupation: Men eFigure 3. Racial/Ethnic Composition by Occupation: Women eTable 5. Lost-Time Injury Incidence Rates per 100 FTE with Adjustment for Demographics and Occupation, by Race/Ethnicity and Sex eFigure 4. Demographic-Adjusted Occupation-Specific Risk Differences between Black non-Hispanic Workers and White non-Hispanic Workers, Men and Women Pooled eFigure 5. Demographic-Adjusted Occupation-Specific Risk Differences between Hispanic Workers and White non-Hispanic Workers, Men and Women Pooled eFigure 6. Demographic-Adjusted Occupation-Specific Risk Differences between Asian/Pacific Islander non-Hispanic Workers and White non-Hispanic Workers, Men and Women Pooled eFigure 7. Demographic-Adjusted Occupation-Specific Risk Differences between Black non-Hispanic Workers and White non-Hispanic Workers, Men eFigure 8. Demographic-Adjusted Occupation-Specific Risk Differences between Hispanic Workers and White non-Hispanic Workers, Men eFigure 9. Demographic-Adjusted Occupation-Specific Risk Differences between Asian/Pacific Islander non-Hispanic Workers and White non-Hispanic Workers, Men eFigure 10. Demographic-Adjusted Occupation-Specific Risk Differences between Black non-Hispanic Workers and White non-Hispanic Workers, Women eFigure 11. Demographic-Adjusted Occupation-Specific Risk Differences between Hispanic Workers and White non-Hispanic Workers, Women eFigure 12. Demographic-Adjusted Occupation-Specific Risk Differences between Asian/Pacific Islander non-Hispanic Workers and White non-Hispanic Workers, Women eTable 6. Sensitivity Analysis: Lost-Time Injury Results Using Winsorized Rates eTable 7. Sensitivity Analysis: Excluding Small Cells eTable 8. Sensitivi [file jamahealthforum-e253495-s001.pdf]

Supplemental Online Content

Dworsky M, Boden LI, Chase EC, et al. Racial and ethnic disparities in occupational health. *JAMA Health Forum*. 2025;6(9):e253495. doi:10.1001/jamahealthforum.2025.3495

eMethods
eTable 1. Rates of Missing Data Before Multiple Imputation
eTable 2. ACS Sample Construction
eTable 3. ACS Sample Construction
eTable 4. Denominator Summary Statistics and Marginal Probabilities
eFigure 1. Racial/Ethnic Composition by Occupation: Total
eFigure 2. Racial/Ethnic Composition by Occupation: Men
eFigure 3. Racial/Ethnic Composition by Occupation: Women
eTable 5. Lost-Time Injury Incidence Rates per 100 FTE with Adjustment for Demographics and Occupation, by Race/Ethnicity and Sex
eFigure 4. Demographic-Adjusted Occupation-Specific Risk Differences between Black non-Hispanic Workers and White non-Hispanic Workers, Men and Women Pooled **eFigure 5.** Demographic-Adjusted Occupation-Specific Risk Differences between Hispanic Workers and White non-Hispanic Workers, Men and Women Pooled
eFigure 6. Demographic-Adjusted Occupation-Specific Risk Differences between Asian/Pacific Islander non-Hispanic Workers and White non-Hispanic Workers, Men and Women Pooled
eFigure 7. Demographic-Adjusted Occupation-Specific Risk Differences between Black non-Hispanic Workers and White non-Hispanic Workers, Men
eFigure 8. Demographic-Adjusted Occupation-Specific Risk Differences between Hispanic Workers and White non-Hispanic Workers, Men
eFigure 9. Demographic-Adjusted Occupation-Specific Risk Differences between Asian/Pacific Islander non-Hispanic Workers and White non-Hispanic Workers, Men
eFigure 10. Demographic-Adjusted Occupation-Specific Risk Differences between Black non-Hispanic Workers and White non-Hispanic Workers, Women
eFigure 11. Demographic-Adjusted Occupation-Specific Risk Differences between Hispanic Workers and White non-Hispanic Workers, Women
eFigure 12. Demographic-Adjusted Occupation-Specific Risk Differences between Asian/Pacific Islander non-Hispanic Workers and White non-Hispanic Workers, Women
eTable 6. Sensitivity Analysis: Lost-Time Injury Results Using Winsorized Rates
eTable 7. Sensitivity Analysis: Excluding Small Cells
eTable 8. Sensitivity Analysis: Results Under Alternative Injury Case Definitions
eTable 9. Sensitivity Analysis: Complete Records Analysis
eTable 10. Sensitivity Analysis: Excluding Predominantly Public-Sector Occupations and Temporary/Contract Employees

© Dworsky M et al, *JAMA Health Forum*

Supplement for Racial and Ethnic Disparities in Occupational Health: Evidence from California Workers' Compensation

Numerator Construction: Workers' Compensation Information System

Our data come from the California Workers' Compensation Information System (WCIS). The WCIS is a database maintained by the Division of Workers' Compensation (DWC) in the California Department of Industrial Relations (DIR) that contains information on all workers' compensation claims.[1] Under a data use agreement, the Division of Workers' Compensation in the California Department of Industrial Relations provided us with WCIS data on all claims with dates of injury from 2005-2019.

California law requires workers compensation claims administrators (insurers, self-insured employers, and third-party administrators) to report all new claims to the WCIS. Like workers' compensation claims databases used in other states, the WCIS is a transaction-based Electronic Data Interchange (EDI) database that generally follows the claims standards defined by the International Association of Industrial Accident Boards and Commissions (IAIABC).

Claim administrators in California are required to submit reports to the WCIS reflecting the occurrence of an injury and subsequent developments, such as benefit payments or case closure. This initial report made upon an injury's occurrence is called the first report on injury (FROI). After the FROI is filed, claims administrators are required to submit additional reports (the subsequent report on injury, or SROI) reflecting material changes in the claim's status, such as claim closure and benefit payments.

Under a data use agreement, programmers at the DWC provided the study team with extracts from FROI and SROI linked to injuries occurring during our 2005-2019 study period. Key data elements from the FROI used directly in the analyses reported here included:

– Sex (DN# 53)

– Age at Injury (derived from Employee Date of Birth DN# 52 and Date of Injury DN# 31)

Assignment of Standard Occupation Classification (SOC) codes using the NIOCCS algorithm (discussed below) used two FROI data elements:

– Occupation Description (DN# 60)

[1] More information about the WCIS can be found at https://www.dir.ca.gov/dwc/wcis.htm. Accessed January 23, 2025.

– Industry Code (DN# 25, SIC codes were crosswalked to two-digit NAICS codes, and invalid NAICS codes were set to missing before running NIOCCS)

Estimation of race/ethnicity probabilities using mBIFSG used several additional data elements:

– Employee Name (First Name DN# 44, Middle Name DN# 45, and Last Name DN# 43)

– Employee Address (DN# 46-50 Address, City, State, and Postal code)

Additional data elements from the FROI that were not used in our analysis were also included in our analytic file and used in our multiple imputation process (discussed below). These included:

– Nature, Part of Body, and Cause of Injury Codes (DN# 35-37)

– Date reported to Employer (DN# 40, used to calculate duration between injury date and date claim was reported)

– Date reported to Claim Administrator (DN# 41, used to calculate duration between injury date and date claim was reported)

– Date of Hire (DN# 61, used to calculate job tenure at injury)

– Class Code (DN# 59)

– Weekly wage (derived from DN# 62 Wage and DN# 63 Wage Period variables)

– Region within California (a 10 region classification used within the workers' compensation system, which we derived from the injured worker's ZIP code)

– Self-insured status of claim (DN# 24)

– Claim denial indicator (constructed by DWC programmers to indicate if claim was denied)

The key SROI data elements used in the current study (including the imputation process) were:

– Paid-to-date amounts (constructed by DWC programmers) indicating the cumulative benefit payments and expenses on the claim at the time of data extraction for a number of detailed benefit type codes

– Date of maximum medical improvement (if any, DN# 70)

Lost-Time Case Definition

Benefit payments reported on subsequent reports of injury are identified using “benefit type codes” (BTCs). We defined lost-time cases to include those with positive paid amounts in any the following BTCs:

- Temporary Disability Benefits
  - 050 Temporary Total
  - 070 Temporary Partial
  - 240 Employer Paid
  - 051 Temporary Total Catastrophic
- Temporary Disability Settlements
  - 550 Compromised Temporary Total
  - 570 Compromised Temporary Partial
  - 524 Compromised Employer Paid
  - 551 Compromised Temporary Total Catastrophic
- Permanent Disability Benefits
  - 020 Permanent Total
  - 030 Permanent Partial Scheduled
  - 090 Permanent Partial Disfigurement
  - 021 Permanent Total Supplemental
  - 040 Permanent Partial Unscheduled
- Permanent Disability Settlements
  - 520 Compromised Permanent Total
  - 530 Compromised Permanent Partial Scheduled
  - 590 Compromised Permanent Partial Disfigurement
  - 521 Compromised Permanent Total Supplemental
  - 540 Compromised Permanent Partial Unscheduled
- Other Settlements
  - 500 Unspecified

We excluded fatality cases from our counts of lost-time cases, where fatality cases were defined to include those with paid or settled death benefits (BTC 010 and 510), or with a date of death reported to the WCIS.

Missing Data

SROI records were de-duplicated and paid amounts were aggregated to the groupings of benefit types listed above, so that we obtained a file with one record for each injured worker who had one or more SROI reported. This file was then linked to the FROI using the Jurisdiction Claim Number (JCN), a unique identifier for an injury claim assigned by the WCIS when a FROI is submitted.

After constructing this file, there were several types of missing data which we needed to address prior to being able to calculate the number of injuries in the cells used in our analysis. In particular:

1. Missing race/ethnicity: Race/ethnicity information is not collected on claims; therefore, this information is missing for everyone in the sample. We imputed race/ethnicity using mBIFSG (discussed below).
2. Absence of structured occupation codes: The FROI provides industry codes, but has only a free text description of the worker's occupation that cannot be used directly for statistical analysis. All claimants in the sample are thus missing SOC codes, but have information about their occupation contained in the occupation description field. We imputed SOC codes using the NIOCCS auto-coder (discussed below).
3. Missingness of benefit payments due to low-quality SROI reporters: Compliance with reporting requirements among the nearly 500 claim administrators in the California workers' compensation system is high but imperfect. While reporting of FROI (which record occurrence of an injury and contain most of the variables needed for our analysis) is essentially complete,[2] some claim administrators rarely or never submit SROI. Because the SROI are needed to capture benefit payments, it is necessary to impute benefit payment information needed to identify lost-time claims for cases submitted by the low-quality SROI reporters.
4. Sporadic missingness: The WCIS data exhibit occasional missingness due to incomplete data entry on claim forms, e.g. claimant's sex is not reported, or the industry code provided is invalid. This is the "standard" sort of missing data that we expect to arise in any kind of dataset. We expect this missingness to be missing at random, and it is comparatively minor (an overview of the percent missing by variable and year is provided below).

[2] California Department of Industrial Relations. (2018). Workers' compensation information system (WCIS): Aggregate FROI and SROI Data.

These four missing data challenges were addressed via multiple imputation, using three algorithms. Modified Bayesian Improved First Name-Surname Geocoding (mBIFSG) was used to impute race/ethnicity, NIOSH Industry and Occupation Computerized Coding System (NIOCCS) was used to assign occupation codes for use in our analysis, and Generalized Efficient Regression-Based Imputation with Latent Processes (GERBIL) was used to address both missing benefit payment information due to low-quality SROI reporters and other sporadic missingness.

Imputation of Race/Ethnicity: mBIFSG

To impute race/ethnicity, we use the mBIFSG algorithm, which uses the information in an individual's name and home address (geocoded to the census block group level) to estimate the probability that the individual's self-reported race/ethnicity would be classified in one of six mutually exclusive categories: Black non-Hispanic, White non-Hispanic, Hispanic, Asian/Pacific Islander non-Hispanic, American Indian/Alaska Native non-Hispanic, or Multiple Race non-Hispanic.[3]

Even after running our analytic data through mBIFSG, there are still some individuals with missing race/ethnicity probability vectors because their name and address were missing from the WCIS. This occurred in only 0.4% of cases. These individuals have race/ethnicity probabilities imputed via GERBIL when we address the sporadic missingness, discussed in the next section. Our approach to converting the mBIFSG probability vectors into a multiply imputed race/ethnicity is discussed below after we describe our approach to sporadic missingness and low-quality SROI reporters.

Validity of Estimates Based on mBIFSG

mBIFSG has very high accuracy for the four largest racial/ethnic groups in the US, where accuracy is defined in terms of the algorithm's ability to predict which race and ethnicity an individual would self-report if asked: a recent comparison of predictions to actual self-reports for

---

[3] For more information on mBIFSG and its predecessor algorithm BISG, please see:

Sorbero, ME, R Euller, A Kofner, and MN Elliott. *Imputation of Race and Ethnicity in Health Insurance Marketplace Enrollment Data, 2015-2022 Open Enrollment Periods.* RAND Corporation (Santa Monica, CA: 2022).

Cabreros, I., D. Agniel, S. C. Martino, C. L. Damberg, and M. N. Elliott. "Predicting Race and Ethnicity to Ensure Equitable Algorithms for Health Care Decision Making." *Health Aff (Millwood)* 41, no. 8 (Aug 2022): 1153-59. https://doi.org/10.1377/hlthaff.2022.00095. https://www.ncbi.nlm.nih.gov/pubmed/35914194.

Elliott, Marc N., Kirsten Becker, Megan K. Beckett, Katrin Hambarsoomian, Philip Pantoja, and Benjamin Karney. "Using Indirect Estimates Based on Name and Census Tract to Improve the Efficiency of Sampling Matched Ethnic Couples from Marriage License Data." *Public Opinion Quarterly* 77, no. 1 (2013): 375-84. https://doi.org/10.1093/poq/nft007.

Elliott, Marc N., Peter A. Morrison, Allen Fremont, Daniel F. McCaffrey, Philip Pantoja, and Nicole Lurie. "Using the Census Bureau's Surname List to Improve Estimates of Race/ Ethnicity and Associated Disparities." *Health Services and Outcomes Research Methodology* 9, no. 2 (2009): 69-83. https://doi.org/10.1007/s10742-009-0047-1.

mBIFSG found C-statistics of 0.96 for Hispanic, 0.96 for Asian/Pacific Islander, 0.95 for Black, and 0.94 for White.[4]

The key assumption necessary for mBIFSG to deliver accurate estimates is that conditional on an individual's true (self-reported) race and ethnicity, their name (first name and surname) and the racial or ethnic composition of their block group are statistically independent. Under this assumption, mBIFSG optimally combines (using Bayes' rule) the information about race and ethnicity contained in geographic, first name, and surname information.

A further assumption necessary for unbiased estimation in our setting is that, conditional on an individual's name and block group, the probability of workers' compensation claim filing is independent of the individual's true (self-reported) race/ethnicity. We were unable to evaluate this assumption in our sample, but validation results indicating good performance for the four largest racial/ethnic groups were found for workers' compensation claims in Washington State.[30,31] Our use of the newer mBIFSG algorithm should improve on the accuracy found in that validation study.

Imputation of Occupation (SOC) Codes: NIOCCS

To assign SOC codes based on the free-text occupation descriptions contained in the WCIS, we use the NIOCCS autocoder, an algorithm developed by NIOSH to improve the efficiency and reproducibility of occupation coding. NIOCCS is a neural net trained on a large corpus of occupation and industry descriptions taken from sources including death certificates and responses to the Behavioral Risk Factor Surveillance System (BRFSS) survey. If an individual only has occupation description (but is missing industry), NIOCCS will still generate a vector of SOC code probabilities.

The version of NIOCCS shared with the study team for use in our secure computing environment takes industry codes and free-text occupation descriptions as inputs and returns a vector of probabilities for each individual, which gives a probability distribution over 808 detailed (6-digit) SOC codes. (Some detailed SOC codes are aggregated to a higher level.)

In addition to generating the probabilities that a trained coder would assign a record to each possible SOC code, NIOCCS can also assign some probability mass to an "Insufficient Information" category, indicating that there is not enough information to assign this person to a particular occupation. To make things computationally feasible, we restrict the probability vector to be

[4] Branham, D Keith, Kenneth Finegold, Lucy Chen, Melony Sorbero, Roald Euller, Marc N Elliott, and Benjamin D Sommers. "Trends in Missing Race and Ethnicity Information after Imputation in Healthcare.Gov Marketplace Enrollment Data, 2015-2021." *JAMA Netw Open* 5, no. 6 (2022): e2216715-e15.

length 30, corresponding to the top 30 most likely occupations (potentially including "insufficient information"). For the vast majority (95%) of the sample, the top 30 occupations comprised 90% or more of their probability mass.[5]

Even after running our analytic file through NIOCCS, we still have some individuals who do not have an occupation probability vector, because they were missing the occupation text description. (This affects 0.4% of the analytic sample.) For individuals who had more than 75% of their probability mass in "Insufficient Information"—an issue that affected 10.1% of cases in the analytic sample—we set their probability vector to missing and imputed occupation probabilities at the 2-digit (Major Occupation) level using GERBIL. Our approach to converting NIOCCS probabilities into a multiply imputed occupation (SOC code) is discussed below after we describe our approach to sporadic missingness and low-quality SROI reporters.

Low-Quality SROI Reporters and Sporadic Missingness: GERBIL

We used GERBIL to address the remaining two missing data issues described above: sporadic missingness and missing benefit payment information due to low-quality SROI reporters. We describe GERBIL after providing additional background on the nature and scope of these two missing data problems.

Low-Quality SROI Reporters

Although California law requires claims administrators to file both the first report of injury (FROI) and subsequent report of injury (SROI), a nontrivial number of claims administrators regularly file only the FROI and not the SROI, possibly due to a lack of training, resources, or interest in complying with the state's reporting requirements. The SROI is critical for providing information on the outcome of the claim that is unknown at the time when the FROI is submitted. Most important for us, the SROI is the source of all information about benefit payments, which we use to identify lost-time claims. Failure to address incomplete SROI reporting will thus result in misclassification of lost-time claims as medical-only claims.

We built on methods developed in past work[6] using WCIS data to identify low-quality SROI reporters by calculating the proportion of claims submitted by each claim administrator that had paid or settled indemnity benefits (which requires submission of a SROI). Claims administrators who reported 100 or fewer FROI over our study period were omitted from this analysis and treated as low-quality SROI reporters, but these claim administrators account for only a trivial number of FROI. After examining the distribution of the indemnity benefit receipt rate, we classified claims administrators for which less than 15% of FROI resulted in indemnity payments as low-quality SROI reporters. (In our past work, we found that cutoffs of 10% or 20% indemnity

---

[5] For more information on how NIOCCS works, please see https://csams.cdc.gov/nioccs/About.aspx.

[6] Dworsky, Michael, Stephanie Rennane, and Nicholas Broten, Wage Loss Monitoring for Injured Workers in California's Workers' Compensation System: 2013 Injury Year Findings (First Interim Report). Santa Monica, CA: RAND Corporation, 2018. https://www.rand.org/pubs/research_reports/RR2572.html. Also available in print form.

claims yielded very similar results, as did a requirement based on the percent of cases with any SROI reported.) For context, 60% of FROI in the workers' compensation system with injury dates between 2010 and 2023 had one or more SROI reported, of which 23% had one or more SROI indicating indemnity payments reported.

To prepare the data for imputation of benefit payment information, receipt of benefits (used to ascertain lost-time cases) was coded as missing for all claims from the low-quality SROI reporters. We have some concern that benefit payment information that is missing due to low-quality SROI reporters is not missing completely at random, i.e., that the probability of missingness and the true values of the missing variables may be correlated with some variables used in our analysis. We are concerned about this because large commercial insurers and the quasi-public State Fund, which account for most claims in the system, have high data quality, while smaller self-insured and self-administered plans, including some local government employers, are more likely to be low-quality SROI reporters. However, given the richness of the information contained on the FROI (as described above), we think it is reasonable to assume that benefit payment information that is missing due to low-quality SROI reporters is missing at random after conditioning on the other variables in our dataset (including age, sex, occupation, employer's industry, type of injury, and sub-state region, among other variables).

Overview of GERBIL

We use GERBIL, a sophisticated approach for jointly imputing missing-at-random data, to produce multiply imputed datasets that can be used for our analysis. GERBIL was developed to address theoretical and computational limitations of the more well-known Multiple Imputation with Chained Equations (MICE) approach. Key advantages of GERBIL include the ability to accommodate arbitrary variable types (including binary, categorical, and other variables with restricted ranges) without requiring variables to be normally distributed, as well as formulation of the data generating process that guarantees existence of a joint probability distribution over the included variables. MICE, in contrast, imposes stronger parametric restrictions on the included variables but can yield results that are inconsistent with any possible joint distribution, which can lead to instability in the resulting imputation datasets. GERBIL specifies all variables as functions of jointly normal latent variables, using suitable transformations (such as copulas for continuous variables) where needed. Details on these transformations are given in Robbins et al. (2013) and Robbins (2014).[7]

[7] Robbins, MW, SK Ghosh, and JD Habiger. "Imputation in High-Dimensional Economic Data as Applied to the Agricultural Resource Management Survey." *Journal of the American Statistical Association* 108, no. 501 (2013): 81-95.

Robbins, MW. "The Utility of Nonparametric Transformations for Imputation of Survey Data." *Journal of Official Statistics* 30, no. 4 (2014): 675-700.

After transformation, GERBIL assumes all variables are latently joint multivariate normal, takes imputation draws from this joint distribution, and then back-transforms to return all imputed values to the observed scale. Robbins (2024) is the authoritative source on GERBIL and should be consulted for technical details.[8]

GERBIL Implementation Details

We feed the FROI and SROI variables listed above into GERBIL along with the vectors of probabilities returned by NIOCCS and mBIFSG. Most of these variables are not used in our analysis, but are included because they are predictive of variables that are used in our analysis. For example, the nature, cause, and body part of injury (which are reported in essentially all cases) are important predictors of whether the injury will result in disability benefit payments (which is missing on cases reported by low-quality SROI reporters); likewise, the industry, region of California, age, and sex of the worker are likely predictive of race/ethnicity and occupation in the small number of cases where mBIFSG and NIOCCS did not return usable probabilities.

Table A.1 describes the proportion of cases (unique FROI) that were missing data on the key variables used in our analysis. Missingness rates are very low–below 1%–for variables other than the NIOCCS probabilities and benefit amounts (which we set to missing for the low-quality SROI reporters).

eTable 1: Rates of Missing Data Before Multiple Imputation

Variable	% of observations with missing data
Sex	0.03
Age at Injury	0.98
Industry (2-digit NAICS)	0.98
Occupation Description	0.41
NIOCCS Result Probabilities	10.5
mBIFSG Result Probabilities	0.03
Benefit receipt and paid amounts (low-quality SROI reporters)	14.9

The variables that are fed into the algorithm (and multiply imputed by GERBIL when missing) are:

– Binary variables: Worker sex; self-insured status; whether a maximum medical improvement date was reported; and whether the claim was denied

[8] Robbins, MW. "Joint Imputation of General Data." *J Surv Stat Methodol* 12, no. 1 (2024): 183-210.

– Categorical variables: industry (2-digit NAICS code); nature of injury; cause of injury; body part of injury; region of California where employee works; calendar quarter of the injury

– Continuous variables: age at injury, workers compensation insurance premium, pre-injury weekly wage, race/ethnicity probabilities produced by mBIFSG, occupation probabilities produced by NIOCCS

– Semicontinuous variables: duration from injury to claims administrator report; duration from injury to employer report; duration from injury to date of maximum medical improvement; and paid benefit amounts in 9 mutually exclusive categories

Semicontinuous variables are variables that are mostly continuous, but that may have a large point mass at a particular value. For example, the amount of permanent disability benefit received is $0 for individuals who never receive permanent disability—the majority of claims– while for individuals who do receive permanent disability benefits, the benefit amount is positive and highly skewed. Similarly, the duration from injury to report of injury are usually semicontinuous, with large point masses at 0, but some claims have a longer lag between injury and report. Finally, there are variables like duration from injury to maximum medical improvement (MMI), which are only observed for individuals who achieve MMI; for individuals who do not, the duration variable is not defined. We accommodate the MMI date in GERBIL by coding individuals who have not achieved MMI as having -99 for this duration value.

Some other variables listed above need further clarification as well.

Workers' compensation premium: We use the workers' compensation pure premium rate for 2020 proposed by the state's workers' compensation actuaries (the Workers' Compensation Insurance Rating Bureau) as a way to parametrize information about the riskiness of occupations (and thus the likely severity of injuries conditional on observed variables) contained in the workers' compensation class code.[9] Workers' compensation class codes are the basic risk classifications used in workers' compensation pricing, and are a required data element on the FROI for fully insured workers' compensation claims. They are also reported voluntarily on many, but not all, self-insured claims.

Benefit Amounts: We aggregate benefit type codes reflecting disability benefits and settlements into five mutually exclusive categories: temporary disability benefits, permanent disability benefits, death benefits, indemnity settlements, and medical settlements. We also include four additional benefit types that are not used in our analysis, but that capture medical costs and thus may contain additional information about injury severity or other worker and injury characteristics: hospital payments, physician payments, other provider payments, and prescription drug payments.

[9] Source: https://www.wcirb.com/sites/default/files/documents/jan2020_approved_pprs_website.xls. Rates for earlier years are not currently available on the WCIRB website, but the relative riskiness of different classifications is unlikely to change dramatically over time.

Occupation Probabilities: Due to computational constraints, we impute the probability of falling into a given 2-digit SOC code (Major Occupation Group) rather than the 3-digit SOC codes (Minor Occupations) used in our analysis. This shortened the occupation probability vector from 76 to 24 elements, which was computationally manageable. Therefore, for individuals who had a missing occupation probability vector, we can only impute their 2-digit SOC code. After GERBIL, but prior to multiple imputation of occupation, we allocated the probability mass in each Major Occupation Group to its constituent Minor Occupations based on the proportions observed among complete records cases (i.e., the 90% of cases that had usable NIOCCS results).

We generated M = 15 imputation datasets from GERBIL, allowing GERBIL's Markov chain Monte-Carlo (MCMC) sampler to run for 150 iterations. Based on our review of trace plots, comparison of imputed and observed mean values, and other MCMC diagnostics, we judged that 150 iterations was enough for the chains to converge. Imputation was conducted separately in each of our 15 years of claims data, building additional flexibility into the imputation process.

Multiple Imputation of Race/Ethnicity and Occupation Codes

After GERBIL, every record in each of our 15 imputation datasets has complete probability vectors for race/ethnicity and occupation. We used these probability vectors to draw race/ethnicity and occupation codes. Because the probabilities generated by GERBIL were constrained to lie between 0 and 1, but we did not impose a constraint requiring them to sum to 1 across categories, we had to renormalize the GERBIL results for race/ethnicity and NIOCCS probabilities. For race/ethnicity, we renormalized the probabilities to sum to 1 for all individuals. For occupation probabilities, we first dropped "Insufficient Information" as a category (removing its probability mass) and then renormalized the probabilities for the remaining occupations to sum to 1.

After renormalizing the occupation and race/ethnicity probability vectors on each imputation, we assign the SOC code and race/ethnicity in each imputation by drawing from a multinomial distribution with probabilities given by the probability vectors. These draws, which were independent of each other and of the GERBIL imputations, serve as each individual's imputed occupation and race/ethnicity in each imputation dataset.

After these steps, data are complete on each imputation and the information we need to construct numerators (how many injuries occurred in a particular category of worker) is ready.

Numerator Aggregation

The steps above give us imputation datasets in which the unit of observation is an individual workers' compensation claim. We need to aggregate this microdata to units of observation corresponding to the denominator estimates (discussed below) that we calculate from the ACS. As described in the paper, we count the number of lost-time injuries over 2005-2019 in cells defined by:

– Age at injury (3 categories): 18-29, 30-49, 50-64

– Sex (2 categories): male, female

– Race/ethnicity (6 categories): Black non-Hispanic, White non-Hispanic, Hispanic, Asian/Pacific Islander non-Hispanic, American Indian/Alaska Native non-Hispanic, or Multiple Race non-Hispanic

– Occupation: 95 categories: 3-digit (Minor Occupation) SOC code.

For sensitivity analyses, we also use alternative case definitions and aggregate them to the cells defined above. These are discussed below when we present sensitivity analysis results.

Sample Flow Table: WCIS

Table A.2 describes the flow of observations from all first reports of injury reported to the WCIS to the population of nonfatal lost-time claims used as the numerator in the analyses reported in the paper.

eTable 2: ACS Sample Construction

Sample	N claims
All FROI 2005-2019	10,142,564
exclude CA non-resident	9,875,761
exclude ages outside 18-64	9,579,125
exclude occupations outside civilian SOC structure	9,556,949
exclude FROI that are not lost-time injuries	2,577,593
exclude fatal lost-time injuries	2,574,881

"Occupations outside civilian SOC structure " refer to a small set of SOC pseudo-codes that can be returned by NIOCCS, but that are not included in the official SOC structure (and thus are not represented among the civilian workforce in the ACS, preventing us from accurately estimating denominators.) In addition to military occupations, NIOCCS can return codes for "homemaker," "volunteer," "retired," "students," and "did not work." We note that some workers who might be accurately assigned to these categories (such as student employees and volunteer firefighters) are covered by the California workers' compensation system. However, because these occupations are not represented in the ACS, we cannot include them in our analysis of incidence rates because denominators for them cannot be defined.

Denominator Construction: American Community Survey

In addition to numerators (how many injuries occurred in a particular category of worker), we also need denominators: how many workers in a particular category were at risk of injury?

To generate these denominators, we use data from the American Community Survey (ACS). We use ACS data from 2005-2019 (corresponding to our years of claims data) from individuals who live in California. We then further restrict the sample to individuals who work in California, are neither self-employed nor federal employees, who are ages 18-64, are not “Other race” (since none of our numerators consider other race, which is not one of the BISG race/ethnicity categories), and do not report a military occupation. After making sample exclusions, we had a sample of 1,913,064 ACS respondents between 2005-2019. The impact of these exclusions is shown in Table A.3:

eTable 3 ACS Sample Construction

Exclusion	Number of ACS Respondents	Weighted Population Size
Starting Sample: All Residents of CA	4,150,173	427,649,870
Restricting to Those Who Work in CA	2,379,822	253,107,729
Removing Self-Employed (Not Incorporated) Workers	2,176,671	232,193,748
Removing Self-Employed (Incorporated) Workers	2,089,918	223,805,452
Removing Federal Government Employees	2,020,788	216,946,802
Removing Unpaid Family Workers	2,016,110	216,499,456
Restricting to Ages 18-64	1,917,463	207,866,249
Removing Other Race Individuals	1,913,072	207,320,589
Removing Individuals from Military Occupations	1,913,064	207,319,945
Final ACS Sample Size	1,913,064	207,319,945

ACS Estimation

As with the numerators, we need to count the number of full-time equivalent workers in each stratified cell based on age, sex, race/ethnicity, and occupation. First, we calculate the annual hours worked for each ACS respondent by multiplying their report on their “usual hours worked in a week” by the number of weeks they worked in the last year. Note that the number of weeks worked in the last year was intervalled (not continuous) for the ACS in 2008-2018; for respondents from these years, we use the midpoint of the interval as the estimate for how many weeks they worked in the last year. After calculating the annual hours worked for each respondent, we calculate the total annual hours worked in each stratification cell, adjusting for the ACS weights. We divide the total annual hours worked in each stratification cell by 2000 to obtain full-time equivalents.

Table A.4 (next page) reports the age and sex breakdown by race/ethnicity in the California workers' compensation-covered workforce over 2005-2019.

eTable 4: Denominator Summary Statistics and Marginal Probabilities

All workers	White		Black		Hispanic		API		AIAN		Multirace		Total	
	N FTE	%	N FTE	%	N FTE	%	N FTE	%	N FTE	%	N FTE	%	N FTE	%
Sex														
Female	2,350,544	44	335,040	51	1,893,036	40	928,952	48	19,775	49	126,400	48	5,653,747	44
Male	2,951,403	56	321,617	49	2,886,983	60	1,023,341	52	20,980	51	136,662	52	7,340,987	56
Age group														
18-29	1,028,386	19	140,139	21	1,400,002	29	359,715	18	8,528	21	81,107	31	3,017,877	23
30-49	2,546,309	48	336,799	51	2,514,264	53	1,089,890	56	19,845	49	133,396	51	6,640,504	51
50-64	1,727,252	33	179,719	27	865,753	18	502,688	26	12,381	30	48,559	18	3,336,352	26

Men	White		Black		Hispanic		API		AIAN		Multirace		Total	
	N FTE	%	N FTE	%	N FTE	%	N FTE	%	N FTE	%	N FTE	%	N FTE	%
Age group														
18-29	548,920	19	69,954	22	830,785	29	186,099	18	4,472	21	40,489	30	1,680,718	23
30-49	1,471,382	50	166,029	52	1,546,263	54	584,078	57	10,423	50	71,346	52	3,849,521	52
50-64	931,101	32	85,634	27	509,936	18	253,164	25	6,085	29	24,827	18	1,810,747	25

Women	White		Black		Hispanic		API		AIAN		Multirace		Total	
	N FTE	%	N FTE	%	N FTE	%	N FTE	%	N FTE	%	N FTE	%	N FTE	%
Age group														
18-29	479,466	20	70,185	21	569,217	30	173,616	19	4,057	21	40,618	32	1,337,159	24
30-49	1,074,927	46	170,770	51	968,001	51	505,812	54	9,422	48	62,050	49	2,790,983	49
50-64	796,151	34	94,084	28	355,818	19	249,523	27	6,296	32	23,732	19	1,525,605	27

Source: authors' calculations, 2005-2019 ACS. Numbers of FTE reported in table are divided by 15 to reflect yearly averages.

Estimation Details

To assess the effect of race/ethnicity on the risk of occupational injury, we fit a series of Poisson regressions. The first model, which measures the crude association between race/ethnicity and occupational injury risk, was of the form:

$$\log E(injuries_c \mid race/ethnicity_c) = \beta_0 + \beta_1 race/ethnicity_c + \log \mathrm{FTE}_c \qquad (1)$$

i.e. a Poisson regression using the number of injuries in a given stratification cell c as the outcome, conditional on the race/ethnicity of the workers in cell c and the number of full-time equivalents (FTE) in cell c as the offset. Note that $\exp \beta_1$ gives the occupational injury risk ratio between workers of a given race/ethnicity and non-Hispanic White workers (the reference category).

To further disentangle the effect of race/ethnicity from other confounders, we then fit a demographic-adjusted version of Model (1):

$$\log E(injuries_c \mid race/ethnicity_c, sex_c, age_c) = \beta_0 + \beta_1 race/ethnicity_c + \beta_2 sex_c + \beta_3 age_c + \log \mathrm{FTE}_c \quad (2)$$

Finally, to assess the effect of occupation, we fit a model that adjusted for it:

$$\log E(injuries_c \mid race/ethnicity_c, sex_c, age_c, occupation_c) = \beta_0 + \beta_1 race/ethnicity_c + \beta_2 sex_c + \beta_3 age_c + \beta_4 occupation_c + \log \mathrm{FTE}_c \qquad (3)$$

We used a pseudo-maximum likelihood estimator with heteroskedasticity-robust standard errors, which allows consistent estimation and valid inference in the presence of overdispersion and other sources of arbitrary heteroskedasticity as long as the conditional expectation of the outcome given the explanatory variables is correctly specified.

After fitting all models and calculating predicted rates and risk differences adjusted for included covariates, estimates from the 15 imputed datasets were pooled using Rubin's Rules (Rubin 1987).[10] Additional details on the analysis can be found in the Appendix. Analyses were carried out using Stata version 18.0 and R version 4.4.1.

---

[10] Rubin D. Multiple imputation for nonresponse in surveys. John Wiley & Sons, Inc.; 1987.

Post-Estimation: Adjusted Rates, Risk Differences, Inference, and Hypothesis Testing

Using the results of each of these models, we calculated an expected number of occupational injuries per 100 FTEs as if all workers were non-Hispanic White. That is, we calculated the average predicted incidence rate in the White non-Hispanic subsample, and then manipulated the race/ethnicity variable to obtain (counterfactual) adjusted rates for the other racial/ethnic groups. Risk differences were calculated as the difference between each racial/ethnic group's adjusted rate and the White non-Hispanic rate.

Variance estimation for the Poisson regressions is done using heteroskedasticity-robust standard errors, which accommodate overdispersion as well as other arbitrary forms of heteroskedasticity. Standard errors for post-estimation quantities like incidence rate ratios, adjusted incidence rates, and risk differences are calculated using the delta method.

In the paper, we interpret the change in the risk difference between the age/sex-adjusted model and the occupation-adjusted model as the component of the age/sex-adjusted disparity that is attributable to occupational segregation. We test the hypothesis that occupational segregation contributes to the disparity in injury risk by conducting a Wald test for equality of the occupation-adjusted and age/sex-adjusted coefficients (which correspond to the adjusted difference in each racial/ethnic group's injury rate from the White non-Hispanic rate). Hypothesis testing across equations was done using Stata's suest command (which effectively stacks the estimating equations from each model into a seemingly unrelated regression objective function) with standard errors clustered at the cell level to account for covariance of the estimates across equations.

Subgroup Analyses and Stratification

Because we saw evidence of intersectional associations between race and sex, we also fit sex-stratified variants of models (1)-(3). We then calculated the crude and adjusted disparities between race/ethnicity groups separately for men and women.

Finally, to further explore the effect of occupation, we fit occupation-stratified variants of Models (1) and (2) and calculated the crude and demographic-adjusted racial/ethnic disparity in occupational injury risk within each occupation. We also test equality of male and female occupation-adjusted disparities by testing equality of the regression coefficients across models as described above.

Accounting for Multiple Imputation

All analyses described above were conducted within a framework of multiple imputation. On each of our M =15 imputations, we carried out the analysis. We then pooled the results from each of the M =15 imputations according to Rubin’s Rules, i.e. took the mean across the 15 imputations as our point estimate and calculated the variance in a way that reflected the additional

uncertainty introduced by the imputation procedure.[11] For all estimates/statistical quantities described above, pooling was the last step of the estimation process.

Supplementary Results

Methods for Exhibit 3

Exhibit 3 in the paper illustrates the association between injury risk and the racial/ethnic distribution of different occupations. The X-axis of the figure gives the crude injury rate per 100 FTE of each occupation. Note that this crude injury rate is the overall injury rate – not stratified by race/ethnicity. The crude injury rate is the average of the rates calculated in each of the 15 imputations, with the numerator—the number of lost time injuries—coming from the imputed WCIS data, and the denominator—the number of FTEs at risk—coming from the ACS, without any imputation.

The y-axis of the figure gives the percent of the FTEs in that occupation that belong to the race/ethnicity group named at the top of each panel, e.g. what percent of FTEs in that occupation are Black non-Hispanic, for the top right panel. This is calculated from the ACS data and does not rely on imputation. The size of the points in the scatterplot indicates the number of FTEs in that occupation.

Finally, a line of best fit is shown on each panel of the plot, with the intercept and slope of the line printed in red font at the top right of each panel. This line of best fit comes from regressing the percent of FTEs belonging to each race/ethnicity on the crude injury rate, separately for each race/ethnicity, in a weighted least squares regression that weights by the number of FTEs in the occupation.

Additional Figures Documenting Occupational Segregation

eFigures 1, 2, and 3 present the racial/ethnic composition of occupations in California over 2005-2019 including the two smaller racial/ethnic groups omitted from Exhibit 2 in the paper. Occupations are shown in descending order of the White non-Hispanic employment share.

[11] Rubin, DB. *Multiple Imputation for Nonresponse in Surveys.* New York, NY: John Wiley & Sons, Inc., 1987.

eFigure 1: Racial/Ethnic Composition by Occupation: Total

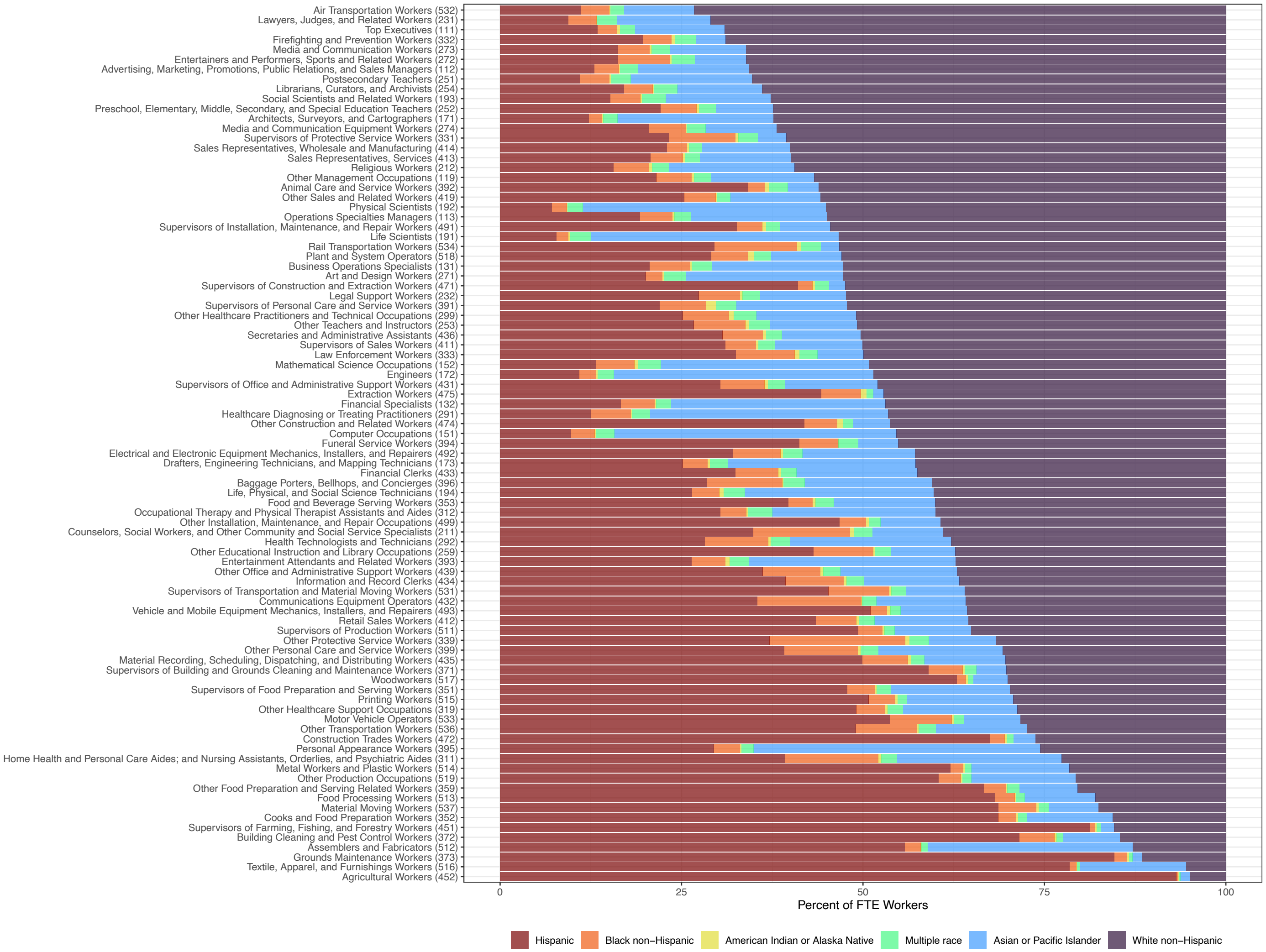

Source: Authors' calculations, 2005-2019 ACS.

eFigure 2: Racial/Ethnic Composition by Occupation: Men

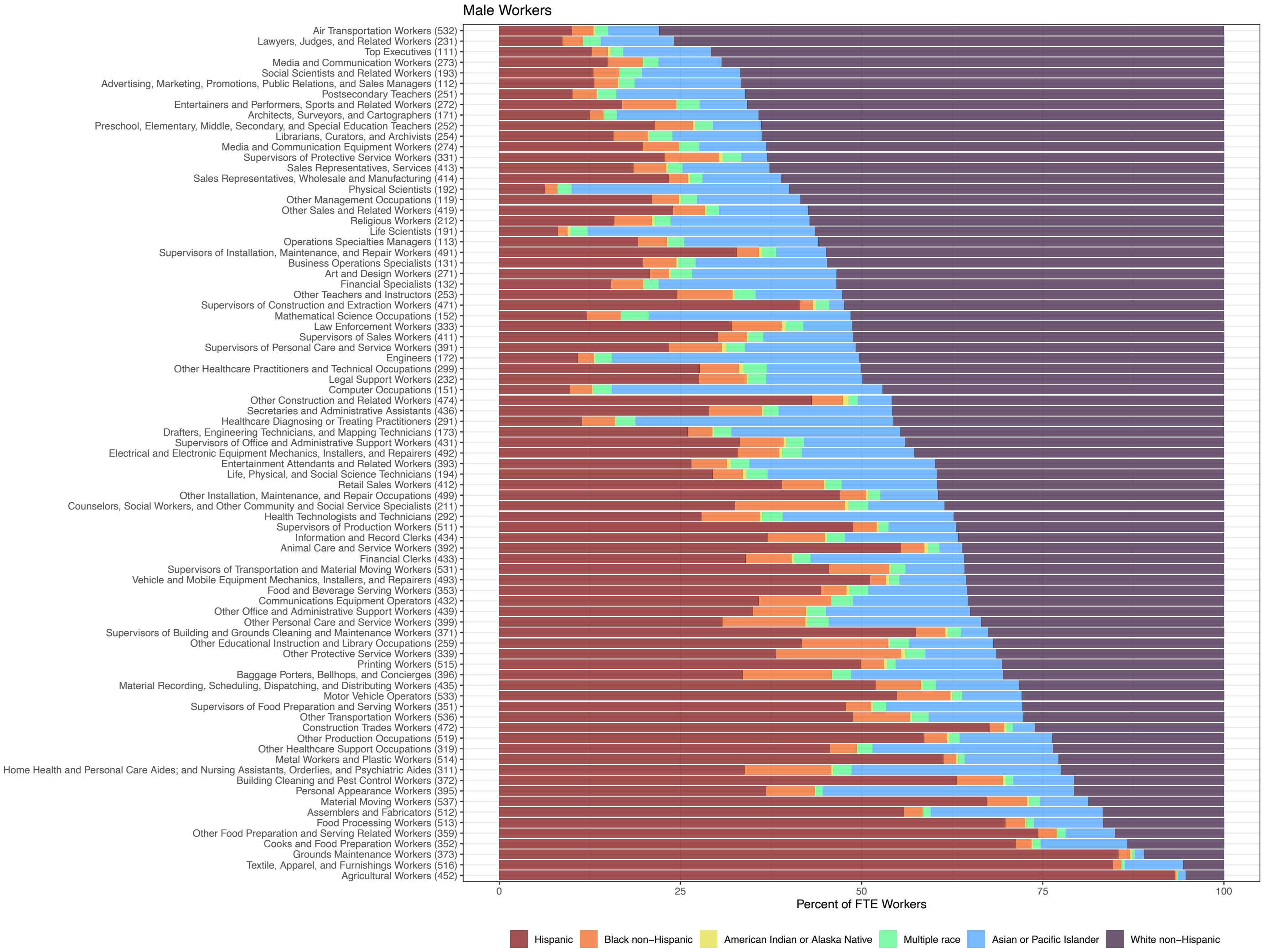

Source: Authors' calculations, 2005-2019 ACS.

eFigure 3: Racial/Ethnic Composition by Occupation: Women

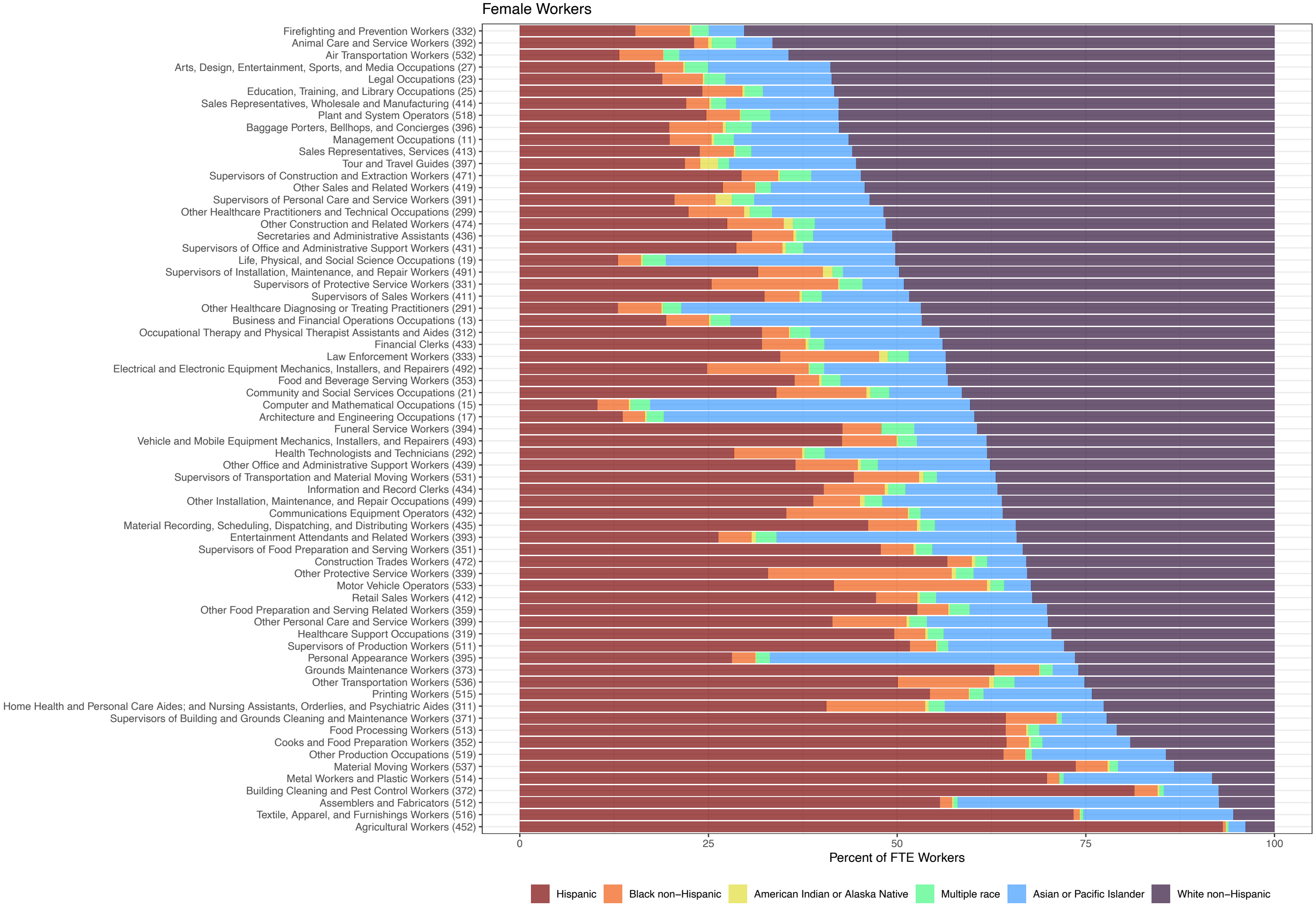

Source: Authors' calculations, 2005-2019 ACS.

Regression Tables for Main Models

Table A.5 shows the estimates underlying Figure 1 in the paper, which shows the findings from our main regression specification.

eTable 5: Lost-Time Injury Incidence Rates per 100 FTE with Adjustment for Demographics and Occupation, by Race/Ethnicity and Sex

Sample	All Workers	All Workers	All Workers	Men	Men	Men	Women	Women	Women
	Unadjusted?	Age/ Sex- Adjusted?	Occupation- Adjusted	Unadjusted?	Age/ Sex- Adjusted?	Occupation- Adjusted	Unadjusted?	Age/ Sex- Adjusted?	Occupation- Adjusted
	Y			Y			Y		
		Y	Y		Y	Y		Y	Y
			Y			Y			Y
**Adjusted Incidence Rates**									
White	1.00	1.00	1.00	1.08	1.08	1.08	0.90	0.90	0.90
	(0.07)	(0.07)	(0.02)	(0.12)	(0.12)	(0.03)	(0.07)	(0.07)	(0.02)
	[0.86, 1.14]	[0.86, 1.14]	[0.95, 1.04]	[0.84, 1.31]	[0.84, 1.31]	[1.02, 1.14]	[0.77, 1.03]	[0.77, 1.03]	[0.85, 0.95]
Black	1.74	1.78	1.37	1.67	1.70	1.26	1.80	1.85	1.43
	(0.09)	(0.09)	(0.04)	(0.14)	(0.14)	(0.04)	(0.13)	(0.13)	(0.06)
	[1.55, 1.92]	[1.60, 1.97]	[1.29, 1.45]	[1.40, 1.94]	[1.43, 1.97]	[1.18, 1.34]	[1.55, 2.05]	[1.59, 2.10]	[1.32, 1.55]
Hispanic	1.90	2.03	1.29	1.96	2.07	1.31	1.82	2.00	1.31
	(0.10)	(0.10)	(0.03)	(0.13)	(0.13)	(0.04)	(0.15)	(0.15)	(0.04)
	[1.70, 2.10]	[1.84, 2.23]	[1.23, 1.36]	[1.70, 2.21]	[1.80, 2.33]	[1.24, 1.38]	[1.52, 2.12]	[1.71, 2.29]	[1.23, 1.40]
API non-Hispanic	0.63	0.64	0.64	0.62	0.63	0.69	0.63	0.65	0.58
	(0.05)	(0.05)	(0.02)	(0.08)	(0.08)	(0.03)	(0.05)	(0.05)	(0.02)
	[0.53, 0.72]	[0.54, 0.74]	[0.60, 0.68]	[0.46, 0.78]	[0.48, 0.79]	[0.63, 0.75]	[0.53, 0.74]	[0.55, 0.75]	[0.54, 0.63]
**Risk Difference Relative to White**									
Black	0.74	0.78	0.37	0.59	0.62	0.19	0.90	0.95	0.53
	(0.12)	(0.12)	(0.05)	(0.18)	(0.18)	(0.05)	(0.14)	(0.15)	(0.06)
	[0.51, 0.97]	[0.55, 1.01]	[0.28, 0.46]	[0.24, 0.95]	[0.27, 0.98]	[0.08, 0.29]	[0.62, 1.18]	[0.66, 1.24]	[0.41, 0.66]
Hispanic	0.90	1.04	0.30	0.88	0.99	0.23	0.92	1.10	0.41
	(0.12)	(0.13)	(0.04)	(0.18)	(0.18)	(0.05)	(0.17)	(0.16)	(0.05)
	[0.66, 1.15]	[0.79, 1.28]	[0.22, 0.38]	[0.53, 1.23]	[0.63, 1.35]	[0.14, 0.33]	[0.59, 1.24]	[0.79, 1.42]	[0.31, 0.52]
API non-Hispanic	-0.37	-0.36	-0.36	-0.46	-0.44	-0.39	-0.27	-0.25	-0.32
	(0.09)	(0.09)	(0.03)	(0.14)	(0.15)	(0.04)	(0.08)	(0.08)	(0.03)
	[-0.54, -0.20]	[-0.53, -0.18]	[-0.42, -0.30]	[-0.74, -0.17]	[-0.73, -0.16]	[-0.47, -0.31]	[-0.43, -0.10]	[-0.42, -0.09]	[-0.38, -0.25]
N (stratification cells)	2,230	2,230	2,230	1,132	1,132	1,132	1,098	1,098	1,098

NOTES: Authors' calculations, 2005-2019 WCIS and ACS. "API" = Asian/Pacific Islander. "FTE" = full-time equivalent. Heteroskedasticity-robust standard errors are in parentheses, and 95% confidence intervals are in brackets. Table reports predicted incidence rates and risk differences from Poisson regression models for lost-time injury counts. Estimates based on 15 multiply imputed datasets. The number of FTE workers (in hundreds) is used as an exposure term (i.e., ln(FTE / 100) is included with the coefficient constrained to equal one. All models include an intercept and indicators for the four race/ethnicity categories shown here (AIANNH and MNH were excluded from the sample). Demographic-adjusted models also include indicators for sex (excluded category = men), age (excluded category = ages 18-29), and sex-age interactions in models for all workers. Demographic-adjusted models stratified by sex include only age category indicators. Demographic and Occupation-adjusted models also include indicators for 95 occupation categories (excluded category = top executives (SOC 11-1000)). Adjusted incidence rates are predicted injury rates per 100 FTE under the distribution of included covariates observed for White workers. Risk Differences for each group are differences between the adjusted rate and the rate observed for White workers.

Full regression results from the regression specifications underlying eTable 5 can be found in the Incidence Disparities Supplement Workbook included as Supplement 2 in the tab titled "full regression tables": they are omitted from this document due to space constraints.

Occupation-Specific Incidence Rates and Within-Occupation Disparity Estimates

In the excel workbook contained in the supplement, we provide data on FTE employment, the number of lost-time injuries, and the unadjusted injury incidence rate at the race/ethnicity by occupation level. These estimates, which are constructed by aggregating over the cells in our analytic file and (for injury counts and rates) averaging the injury counts across imputation datasets, may be of interest to researchers and policymakers. Statistics by race/ethnicity and occupation for all workers are contained in the "rates by occ total" tab, while statistics by race/ethnicity, sex, and occupation are contained in the "rates by occ by sex" tab.

To protect confidentiality and avoid reporting potentially unreliable estimates, injury counts and rates are suppressed for occupation-race/ethnicity-sex cells that either have fewer than 1500 FTE workers over 2005-2019 (or 100 per year on average), or that have fewer than 11 lost-time injuries over 2005-2019. FTE employment for these cells is reported since FTE employment was derived from the public-use ACS.

The incidence rates contained in the workbook are unadjusted. To obtain estimates of within-occupation disparities that adjust for demographic (age and sex differences) between groups, we also estimated Poisson regression models using the specification given in Equation (2) above, but stratified on occupation to obtain occupation-specific disparity estimates. Within-occupation disparities were estimated for all workers, for men, and for women. Estimates for all workers were used to create Figure 3 in the paper.

eFigures 4 through 12 (starting next page) present forest plots illustrating the demographic-adjusted within-occupation risk differences for each non-White racial/ethnic group, sorted in decreasing order of the risk difference from White non-Hispanic workers. Error bars indicate 95% confidence intervals. These estimates may be of interest to readers who want to understand which occupations have the largest within-occupation disparities in injury rates.

eFigure 4: Demographic-Adjusted Occupation-Specific Risk Differences between Black non-Hispanic Workers and White non-Hispanic Workers, Men and Women Pooled

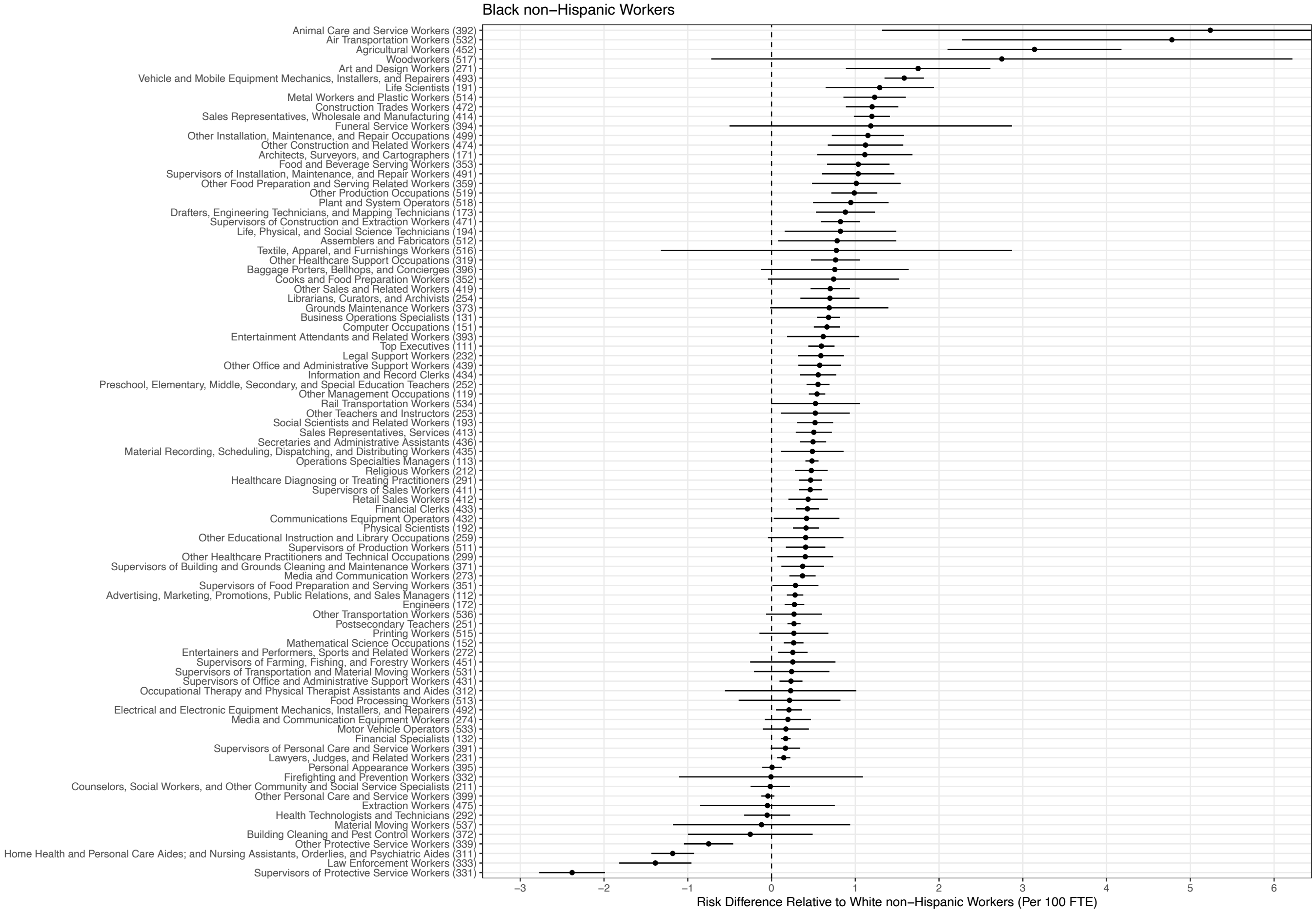

NOTES: Authors' calculations, 2005-2019 WCIS and ACS. Error bars indicate 95% confidence intervals. Estimates based on 15 multiply imputed datasets. The risk difference is adjusted for demographic (age and sex) differences from the White non-Hispanic population using a Poisson regression with the number of FTE workers (in hundreds) included as an exposure term (i.e., ln(FTE / 100) is included with the coefficient constrained to equal one), an intercept, indicators for race/ethnicity categories (WNH is the excluded category; AIANNH and MNH were excluded from the sample), and indicators for sex (excluded category = men), age (excluded category = ages 18-29), and sex-age interactions. Models are stratified by 95 occupation categories.

eFigure 5: Demographic-Adjusted Occupation-Specific Risk Differences between Hispanic Workers and White non-Hispanic Workers, Men and Women Pooled

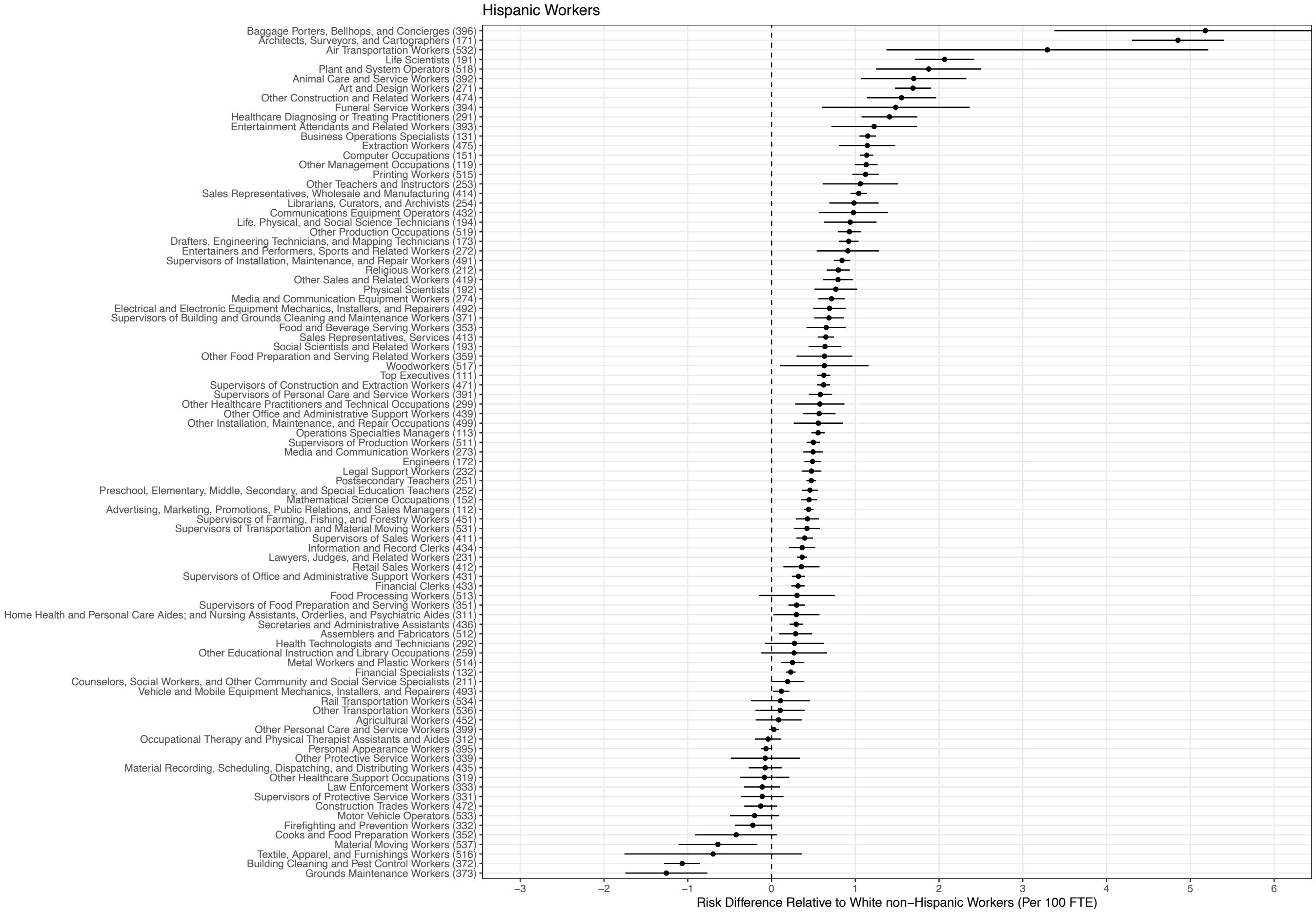

NOTES: Authors' calculations, 2005-2019 WCIS and ACS. Error bars indicate 95% confidence intervals. Estimates based on 15 multiply imputed datasets. The risk difference is adjusted for demographic (age and sex) differences from the White non-Hispanic population using a Poisson regression with the number of FTE workers (in hundreds) included as an exposure term (i.e., ln(FTE / 100) is included with the coefficient constrained to equal one), an intercept, indicators for race/ethnicity categories (WNH is the excluded category; AIANNH and MNH were excluded from the sample), and indicators for sex (excluded category = men), age (excluded category = ages 18-29), and sex-age interactions. Models are stratified by 95 occupation categories.

eFigure 6: Demographic-Adjusted Occupation-Specific Risk Differences between Asian/Pacific Islander non-Hispanic Workers and White non-Hispanic Workers, Men and Women Pooled

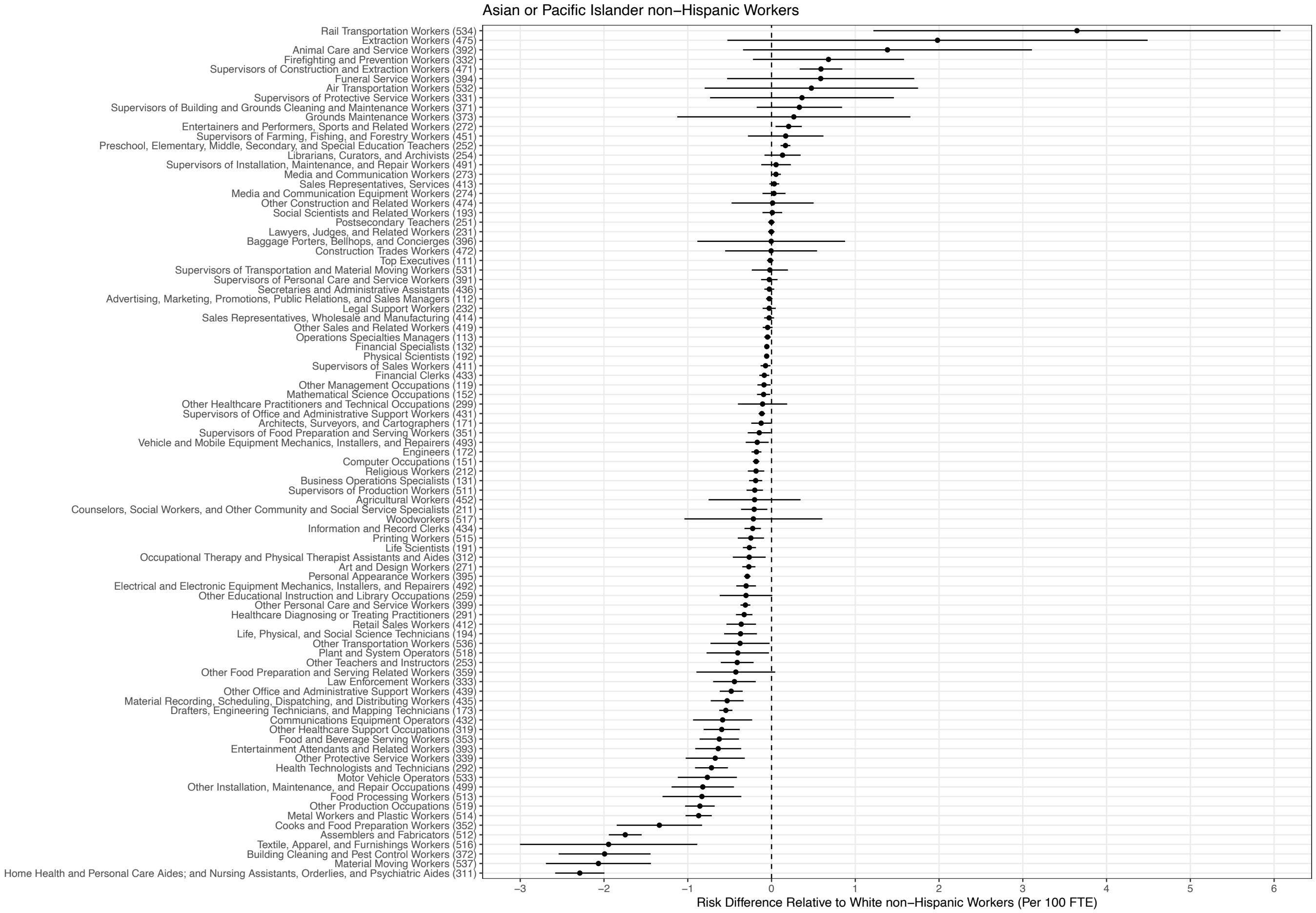

NOTES: Authors' calculations, 2005-2019 WCIS and ACS. Error bars indicate 95% confidence intervals. Estimates based on 15 multiply imputed datasets. The risk difference is adjusted for demographic (age and sex) differences from the White non-Hispanic population using a Poisson regression with the number of FTE workers (in hundreds) included as an exposure term (i.e., ln(FTE / 100) is included with the coefficient constrained to equal one), an intercept, indicators for race/ethnicity categories (WNH is the excluded category; AIANNH and MNH were excluded from the sample), and indicators for sex (excluded category = men), age (excluded category = ages 18-29), and sex-age interactions. Models are stratified by 95 occupation categories.

eFigure 7: Demographic-Adjusted Occupation-Specific Risk Differences between Black non-Hispanic Workers and White non-Hispanic Workers, Men

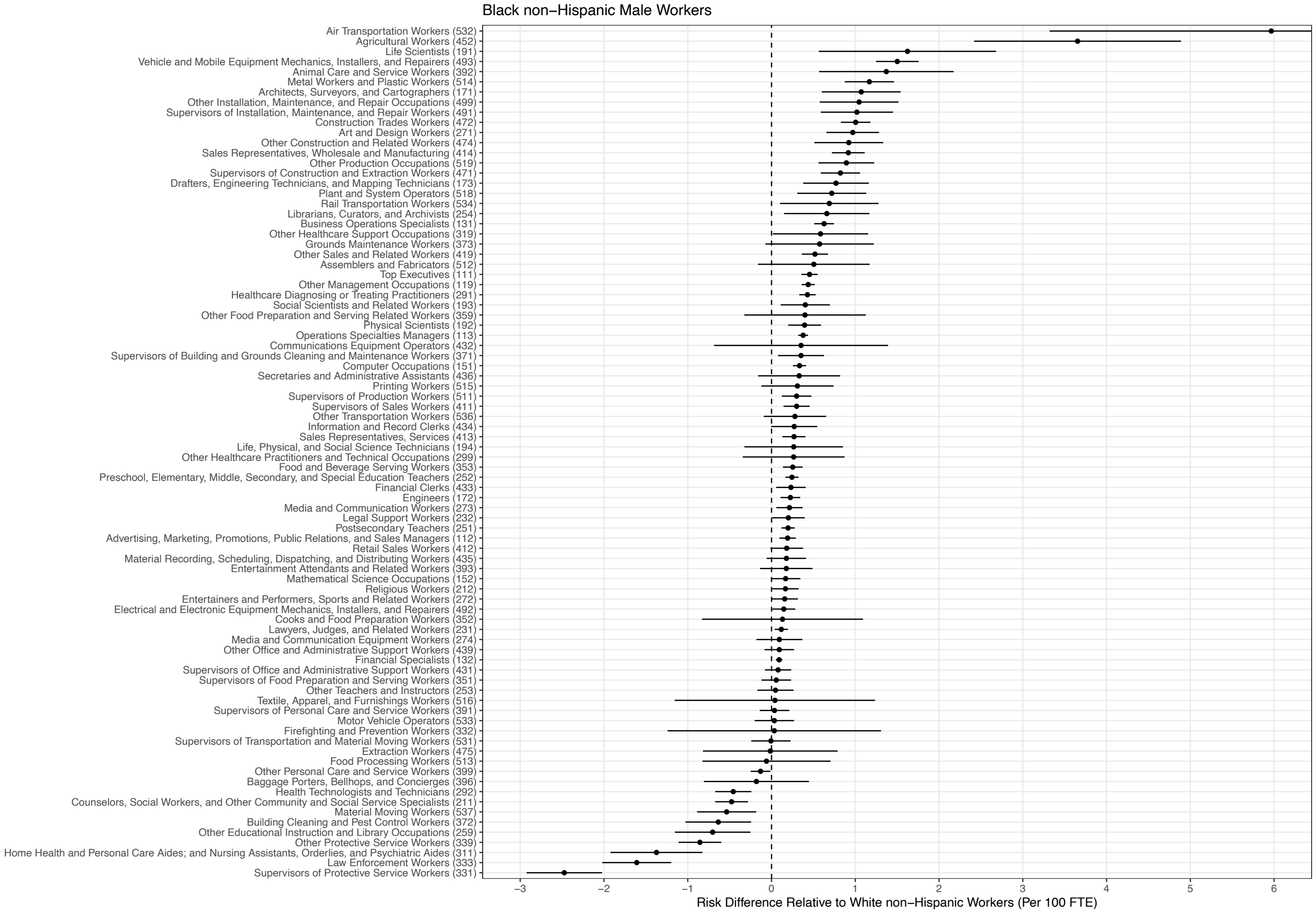

NOTES: Authors' calculations, 2005-2019 WCIS and ACS. Error bars indicate 95% confidence intervals. Estimates based on 15 multiply imputed datasets. The risk difference is adjusted for demographic (age) differences from the White non-Hispanic population using a Poisson regression with the number of FTE workers (in hundreds) included as an exposure term (i.e., ln(FTE / 100) is included with the coefficient constrained to equal one), an intercept, indicators for race/ethnicity categories (WNH is the excluded category; AIANNH and MNH were excluded from the sample), and indicators for age (excluded category = ages 18-29). Models are stratified by 95 occupation categories.

eFigure 8: Demographic-Adjusted Occupation-Specific Risk Differences between Hispanic Workers and White non-Hispanic Workers, Men

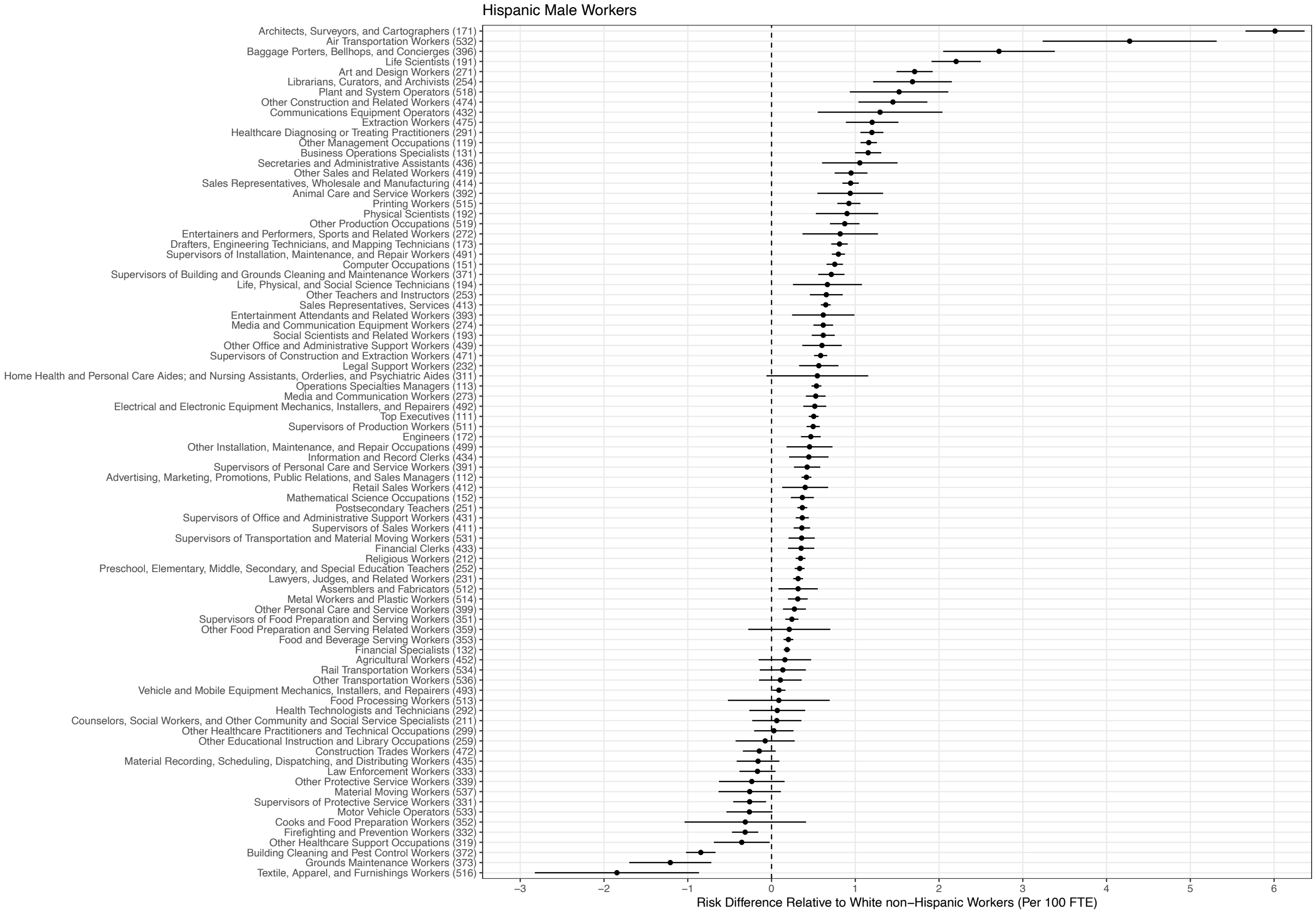

NOTES: Authors' calculations, 2005-2019 WCIS and ACS. Error bars indicate 95% confidence intervals. Estimates based on 15 multiply imputed datasets. The risk difference is adjusted for demographic (age) differences from the White non-Hispanic population using a Poisson regression with the number of FTE workers (in hundreds) included as an exposure term (i.e., ln(FTE / 100) is included with the coefficient constrained to equal one), an intercept, indicators for race/ethnicity categories (WNH is the excluded category; AIANNH and MNH were excluded from the sample), and indicators for age (excluded category = ages 18-29). Models are stratified by 95 occupation categories.

eFigure 9: Demographic-Adjusted Occupation-Specific Risk Differences between Asian/Pacific Islander non-Hispanic Workers and White non-Hispanic Workers, Men

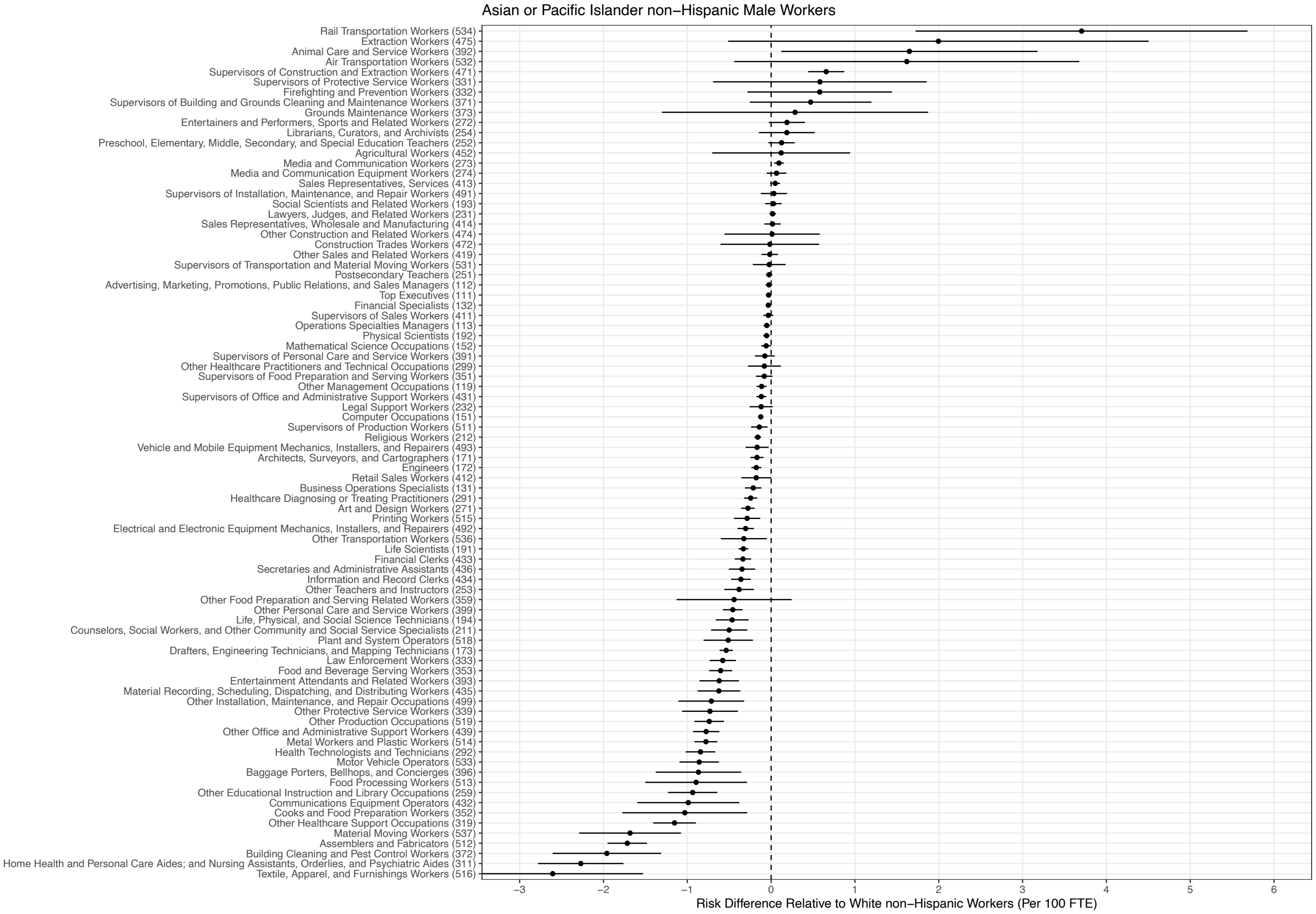

NOTES: Authors' calculations, 2005-2019 WCIS and ACS. Error bars indicate 95% confidence intervals. Estimates based on 15 multiply imputed datasets. The risk difference is adjusted for demographic (age) differences from the White non-Hispanic population using a Poisson regression with the number of FTE workers (in hundreds) included as an exposure term (i.e., ln(FTE / 100) is included with the coefficient constrained to equal one), an intercept, indicators for race/ethnicity categories (WNH is the excluded category; AIANNH and MNH were excluded from the sample), and indicators for age (excluded category = ages 18-29). Models are stratified by 95 occupation categories.

eFigure 10: Demographic-Adjusted Occupation-Specific Risk Differences between Black non-Hispanic Workers and White non-Hispanic Workers, Women

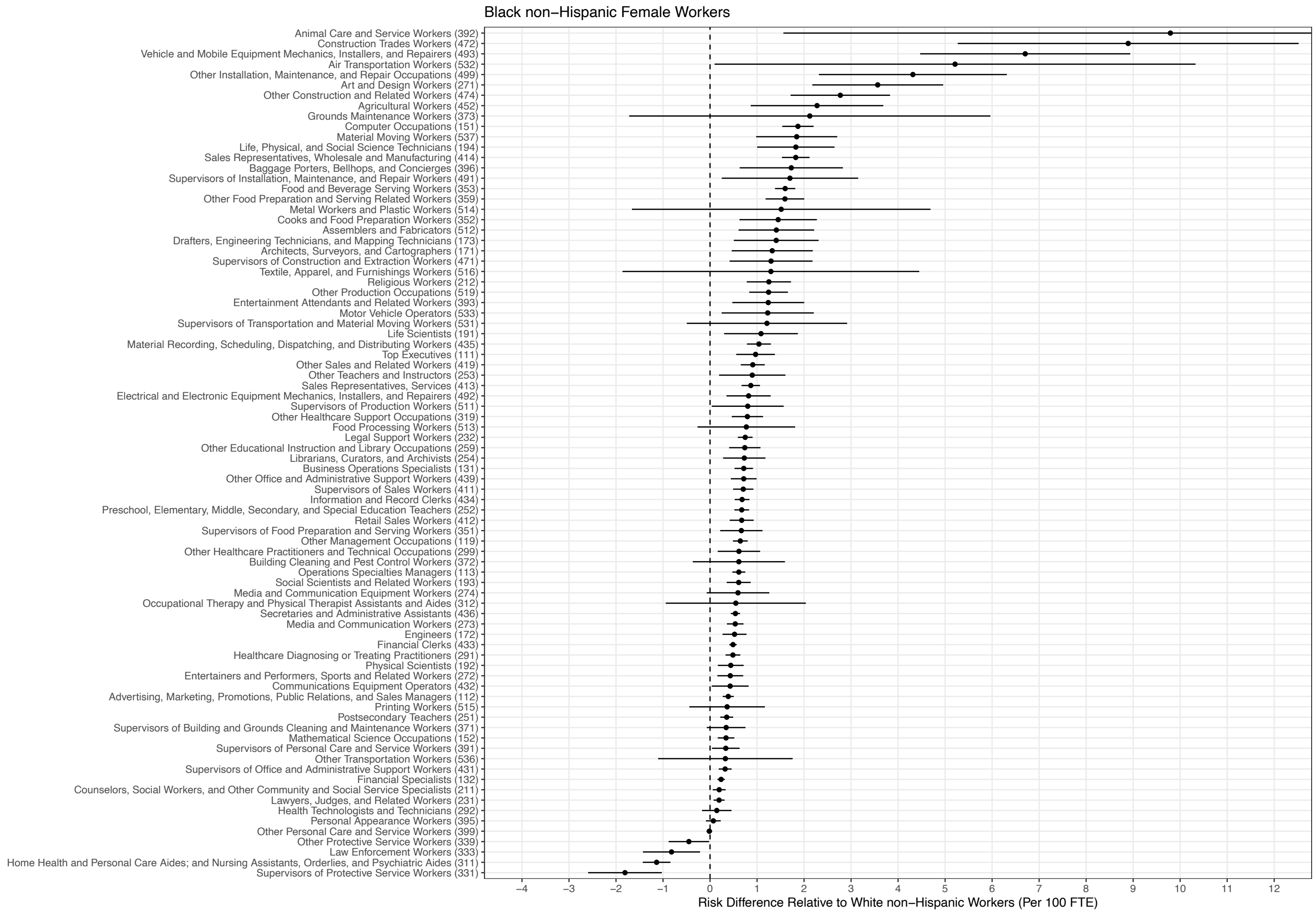

NOTES: Authors' calculations, 2005-2019 WCIS and ACS. Error bars indicate 95% confidence intervals. Estimates based on 15 multiply imputed datasets. The risk difference is adjusted for demographic (age) differences from the White non-Hispanic population using a Poisson regression with the number of FTE workers (in hundreds) included as an exposure term (i.e., ln(FTE / 100) is included with the coefficient constrained to equal one), an intercept, indicators for race/ethnicity categories (WNH is the excluded category; AIANNH and MNH were excluded from the sample), and indicators for age (excluded category = ages 18-29). Models are stratified by 95 occupation categories.

eFigure 11: Demographic-Adjusted Occupation-Specific Risk Differences between Hispanic Workers and White non-Hispanic Workers, Women

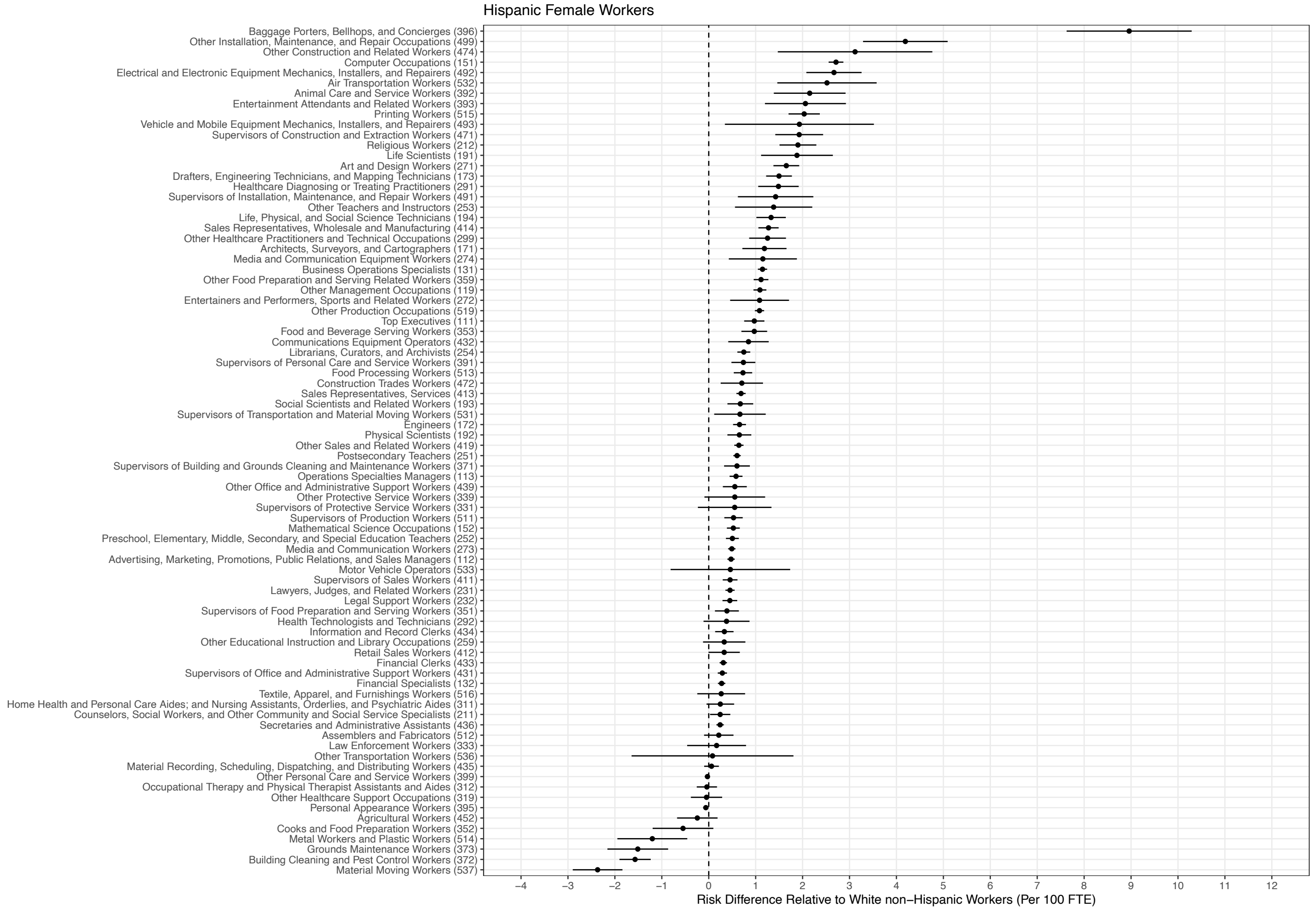

NOTES: Authors' calculations, 2005-2019 WCIS and ACS. Error bars indicate 95% confidence intervals. Estimates based on 15 multiply imputed datasets. The risk difference is adjusted for demographic (age) differences from the White non-Hispanic population using a Poisson regression with the number of FTE workers (in hundreds) included as an exposure term (i.e., ln(FTE / 100) is included with the coefficient constrained to equal one), an intercept, indicators for race/ethnicity categories (WNH is the excluded category; AIANNH and MNH were excluded from the sample), and indicators for age (excluded category = ages 18-29). Models are stratified by 95 occupation categories.

eFigure 12: Demographic-Adjusted Occupation-Specific Risk Differences between Asian/Pacific Islander non-Hispanic Workers and White non-Hispanic Workers, Women

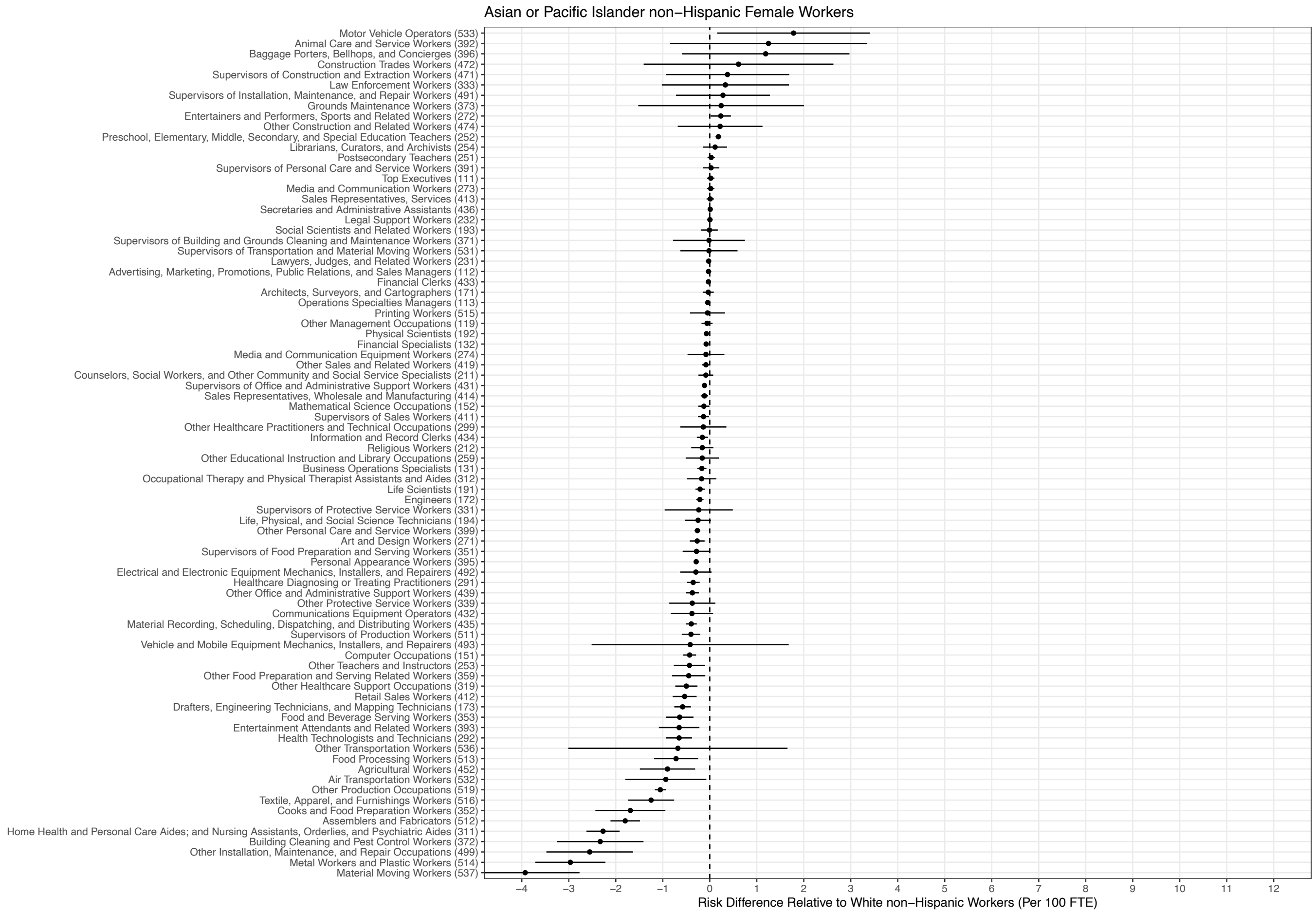

NOTES: Authors' calculations, 2005-2019 WCIS and ACS. Error bars indicate 95% confidence intervals. Estimates based on 15 multiply imputed datasets. The risk difference is adjusted for demographic (age) differences from the White non-Hispanic population using a Poisson regression with the number of FTE workers (in hundreds) included as an exposure term (i.e., ln(FTE / 100) is included with the coefficient constrained to equal one), an intercept, indicators for race/ethnicity categories (WNH is the excluded category; AIANNH and MNH were excluded from the sample), and indicators for age (excluded category = ages 18-29). Models are stratified by 95 occupation categories.

Sensitivity Analyses for Regression Models

eTable 6 shows adjusted rates and risk differences from two sensitivity analyses intended to ensure that our main findings for lost-time injuries are not driven by outliers in the cell-specific rates (which might occur in smaller cells due to sampling error in the ACS denominator estimates or, potentially, inaccuracy in the NIOCCS SOC coding results).

The first three columns winsorize the cell-level rates at the 99th percentile of the distribution observed in the main regression sample. The second three columns winsorize at the 95th percentile. (To be precise, we calculate cell-specific incidence rates, winsorize the rates, and then calculate winsorized counts of injuries by multiplying the winsorized rate by the denominator and rounding to the nearest whole number.) Results from these sensitivity analyses are nearly identical to the main results shown in Exhibit 2 of the paper.

To further guard against possible bias due to outliers, eTable 7 shows a sensitivity analysis that excludes occupations with small cells (defined by having few FTE in the denominator) from the analysis.

eTable 8 shows results using different case definitions. The first three columns of eTable 8 show estimates for lost-time injuries, but excluding approximately 5% of cases that were classified as lost-time injuries on the basis of settlements only. That is, the case definition requires that workers received regular, periodic TD or PD benefits. The second three columns include all workers' compensation claims filed, including medical-only claims that did not result in lost work time or work disability as well as claims that were not paid due to a claim denial. The third three columns include all accepted workers' compensation claims (including both lost-time and medical-only claims). eTable 9 shows two sets of results that exclude claims that were imputed. The first three columns exclude claims from unreliable claim administrators but include other imputed cases. The second three columns exclude all imputed cases. Finally, eTable 10 shows results for different subsamples of workers. The first three columns exclude occupations that are predominantly employed in the public sector (primarily public safety workers and teachers). The second three columns include all occupations, but excludes claims from workers employed by temporary agencies, professional employer organizations, and other labor intermediaries.

While changes in the case definition and the included sample change the magnitudes of the predicted rates and risk differences, none of these sensitivity analyses lead to qualitatively different conclusions from those indicated by our main analysis (Figure 1 in the paper).

eTable 6: Sensitivity Analysis: Lost-Time Injury Results Using Winsorized Rates

Outcome	Lost-Time	Lost-Time	Lost-Time	Lost-Time	Lost-Time	Lost-Time
Winsorized?	p99	p99	p99	p95	p95	p95
	Unadjusted?<br>Y	Age/Sex-Adjusted?<br>Y	Occupation-adjusted<br>Y<br>Y	Unadjusted?<br>Y	Age/Sex-Adjusted?<br>Y	Occupation-adjusted<br>Y<br>Y
**Incidence Rates**						
White Non-Hispanic	1.00	1.00	1.00	1.00	1.00	1.00
	(0.07)	(0.07)	(0.02)	(0.07)	(0.07)	(0.02)
	[0.86, 1.14]	[0.86, 1.14]	[0.95, 1.04]	[0.86, 1.14]	[0.85, 1.14]	[0.95, 1.04]
Black Non-Hispanic	1.74	1.78	1.37	1.72	1.77	1.35
	(0.09)	(0.09)	(0.04)	(0.09)	(0.09)	(0.04)
	[1.55, 1.92]	[1.60, 1.97]	[1.29, 1.45]	[1.54, 1.90]	[1.58, 1.95]	[1.28, 1.43]
Hispanic	1.90	2.03	1.29	1.90	2.03	1.29
	(0.10)	(0.10)	(0.03)	(0.10)	(0.10)	(0.03)
	[1.70, 2.10]	[1.84, 2.23]	[1.23, 1.36]	[1.70, 2.10]	[1.84, 2.23]	[1.23, 1.36]
API Non-Hispanic	0.63	0.64	0.64	0.63	0.64	0.64
	(0.05)	(0.05)	(0.02)	(0.05)	(0.05)	(0.02)
	[0.53, 0.72]	[0.54, 0.74]	[0.60, 0.68]	[0.53, 0.72]	[0.54, 0.74]	[0.60, 0.68]
Risk Difference Relative to White Non-Hispanic						
Black Non-Hispanic	0.74	0.78	0.37	0.72	0.77	0.36
	(0.12)	(0.12)	(0.05)	(0.12)	(0.12)	(0.05)
	[0.51, 0.97]	[0.55, 1.01]	[0.28, 0.46]	[0.49, 0.95]	[0.54, 1.00]	[0.27, 0.45]
Hispanic	0.90	1.04	0.30	0.90	1.03	0.29
	(0.12)	(0.13)	(0.04)	(0.12)	(0.13)	(0.04)
	[0.66, 1.15]	[0.79, 1.28]	[0.22, 0.38]	[0.66, 1.14]	[0.79, 1.28]	[0.21, 0.37]
API Non-Hispanic	-0.37	-0.36	-0.36	-0.37	-0.36	-0.36
	(0.09)	(0.09)	(0.03)	(0.09)	(0.09)	(0.03)
	[-0.54, -0.20]	[-0.53, -0.18]	[-0.42, -0.30]	[-0.54, -0.20]	[-0.53, -0.18]	[-0.42, -0.30]
Number of Observations	2230	2230	2230	2230	2230	2230

NOTES: Authors' calculations, 2005-2019 WCIS and ACS. "API" = Asian/Pacific Islander. Heteroskedasticity-robust standard errors are in parentheses, and 95% confidence intervals are in brackets. Table reports predicted incidence rates and risk differences from Poisson regression models for lost-time injury counts. Estimates based on 15 multiply imputed datasets. "p99" = 99th percentile of incidence rate distribution over race/ethnicity-age-sex-occupation cells. "p95" = 95th percentile of incidence rate distribution.

eTable 7: Sensitivity Analysis: Excluding Small Cells

Outcome	Lost-Time	Lost-Time	Lost-Time	Lost-Time	Lost-Time	Lost-Time	Lost-Time	Lost-Time	Lost-Time
Minimum FTE, 2005-2019	1500	1500	1500	3000	3000	3000	7500	7500	7500
	Unad-justed? Y	Age/Sex-Adjusted? Y	Occupation-Adjusted Y Y	Unad-justed? Y	Age/Sex-Adjusted? Y	Occupation-Adjusted Y Y	Unad-justed? Y	Age/Sex-Adjusted? Y	Occupation-Adjusted Y Y
**Incidence Rates**									
White Non-Hispanic	1.00	1.00	1.00	1.00	1.00	1.00	1.00	1.00	1.00
	(0.07)	(0.07)	(0.02)	(0.07)	(0.07)	(0.02)	(0.07)	(0.07)	(0.02)
	[0.85, 1.14]	[0.85, 1.14]	[0.95, 1.04]	[0.85, 1.14]	[0.85, 1.14]	[0.95, 1.04]	[0.85, 1.14]	[0.85, 1.14]	[0.95, 1.04]
Black Non-Hispanic	1.73	1.78	1.37	1.73	1.78	1.36	1.73	1.78	1.36
	(0.09)	(0.09)	(0.04)	(0.09)	(0.09)	(0.04)	(0.09)	(0.09)	(0.04)
	[1.55, 1.92]	[1.60, 1.97]	[1.29, 1.45]	[1.55, 1.92]	[1.60, 1.96]	[1.28, 1.44]	[1.55, 1.92]	[1.59, 1.96]	[1.28, 1.44]
Hispanic	1.90	2.03	1.29	1.90	2.03	1.29	1.89	2.03	1.28
	(0.10)	(0.10)	(0.03)	(0.10)	(0.10)	(0.03)	(0.10)	(0.10)	(0.03)
	[1.70, 2.09]	[1.83, 2.22]	[1.23, 1.35]	[1.70, 2.10]	[1.84, 2.23]	[1.23, 1.35]	[1.70, 2.09]	[1.83, 2.23]	[1.22, 1.35]
API Non-Hispanic	0.63	0.64	0.64	0.62	0.64	0.63	0.63	0.64	0.63
	(0.05)	(0.05)	(0.02)	(0.05)	(0.05)	(0.02)	(0.05)	(0.05)	(0.02)
	[0.53, 0.72]	[0.54, 0.74]	[0.60, 0.67]	[0.53, 0.72]	[0.54, 0.74]	[0.60, 0.67]	[0.53, 0.72]	[0.54, 0.74]	[0.60, 0.67]
**Risk Difference Relative to White Non-Hispanic**									
Black Non-Hispanic	0.74	0.79	0.37	0.74	0.79	0.37	0.74	0.78	0.36
	(0.12)	(0.12)	(0.05)	(0.12)	(0.12)	(0.05)	(0.12)	(0.12)	(0.05)
	[0.51, 0.97]	[0.55, 1.02]	[0.28, 0.46]	[0.51, 0.97]	[0.55, 1.02]	[0.28, 0.46]	[0.50, 0.97]	[0.55, 1.02]	[0.27, 0.46]
Hispanic	0.90	1.03	0.30	0.90	1.04	0.29	0.90	1.03	0.29
	(0.12)	(0.13)	(0.04)	(0.12)	(0.13)	(0.04)	(0.12)	(0.13)	(0.04)
	[0.66, 1.14]	[0.79, 1.28]	[0.21, 0.38]	[0.66, 1.15]	[0.79, 1.28]	[0.21, 0.37]	[0.65, 1.14]	[0.79, 1.28]	[0.21, 0.37]
API Non-Hispanic	-0.37	-0.36	-0.36	-0.37	-0.36	-0.36	-0.37	-0.36	-0.36
	(0.09)	(0.09)	(0.03)	(0.09)	(0.09)	(0.03)	(0.09)	(0.09)	(0.03)
	[-0.54, -0.20]	[-0.53, -0.18]	[-0.42, -0.30]	[-0.54, -0.20]	[-0.53, -0.18]	[-0.42, -0.30]	[-0.54, -0.20]	[-0.53, -0.18]	[-0.42, -0.30]
Number of Observations	2116	2116	2116	2013	2013	2013	1917	1917	1917

NOTES: Authors' calculations, 2005-2019 WCIS and ACS. "API" = Asian/Pacific Islander. Heteroskedasticity-robust standard errors are in parentheses, and 95% confidence intervals are in brackets. Table reports predicted incidence rates and risk differences from Poisson regression models for lost-time injury counts. Estimates based on 15 multiply imputed datasets. Samples used in estimation restricted to cells with race/ethnicity-age-sex-occupation cells with FTE (over 2005-2019) above minimum thresholds reporteed in column headers.

eTable 8: Sensitivity Analysis: Results Under Alternative Injury Case Definitions

Case Definition	Paid TD/PD	Paid TD/PD	Paid TD/PD	All Claims	All Claims	All Claims	Accepted Claims	Accepted Claims	Accepted Claims
Sex	All Workers	All Workers	All Workers	All Workers	All Workers	All Workers	All Workers	All Workers	All Workers
	Unadjusted?	Age/Sex-Adjusted?	Occupation-Adjusted	Unadjusted?	Age/Sex-Adjusted?	Occupation-Adjusted	Unadjusted?	Age/Sex-Adjusted?	Occupation-Adjusted
	Y			Y			Y		
		Y	Y		Y	Y		Y	Y
			Y			Y			Y
**Incidence Rates**									
White Non-Hispanic	0.96	0.96	0.96	3.87	3.87	3.87	3.47	3.47	3.47
	(0.07) [0.82, 1.10]	(0.07) [0.82, 1.10]	(0.02) [0.92, 1.00]	(0.24) [3.40, 4.34]	(0.24) [3.40, 4.33]	(0.08) [3.70, 4.03]	(0.22) [3.05, 3.90]	(0.21) [3.06, 3.89]	(0.07) [3.33, 3.62]
Black Non-Hispanic	1.65	1.69	1.30	6.14	6.17	4.92	5.32	5.33	4.26
	(0.09) [1.47, 1.82]	(0.09) [1.52, 1.87]	(0.04) [1.22, 1.37]	(0.28) [5.60, 6.69]	(0.28) [5.63, 6.72]	(0.14) [4.65, 5.19]	(0.24) [4.84, 5.79]	(0.24) [4.86, 5.81]	(0.12) [4.03, 4.49]
Hispanic	1.73	1.85	1.18	6.78	6.85	4.76	5.99	5.99	4.16
	(0.09) [1.55, 1.91]	(0.09) [1.67, 2.02]	(0.03) [1.12, 1.24]	(0.31) [6.16, 7.39]	(0.30) [6.25, 7.45]	(0.11) [4.54, 4.99]	(0.27) [5.45, 6.53]	(0.26) [5.47, 6.50]	(0.10) [3.96, 4.36]
API Non-Hispanic	0.59	0.61	0.61	2.65	2.68	2.68	2.38	2.40	2.41
	(0.05) [0.50, 0.69]	(0.05) [0.52, 0.70]	(0.02) [0.57, 0.65]	(0.19) [2.28, 3.02]	(0.18) [2.32, 3.04]	(0.08) [2.53, 2.83]	(0.17) [2.05, 2.71]	(0.17) [2.08, 2.73]	(0.07) [2.28, 2.54]

Risk Difference Relative to White Non-Hispanic

Black Non-Hispanic	0.69	0.73	0.34	2.28	2.31	1.05	1.84	1.86	0.79
	(0.11) [0.47, 0.91]	(0.11) [0.51, 0.95]	(0.05) [0.25, 0.43]	(0.37) [1.56, 3.00]	(0.36) [1.60, 3.02]	(0.16) [0.74, 1.37]	(0.33) [1.20, 2.48]	(0.32) [1.24, 2.49]	(0.14) [0.51, 1.06]
Hispanic	0.77	0.89	0.22	2.91	2.98	0.90	2.52	2.51	0.69
	(0.11) [0.55, 0.99]	(0.12) [0.66, 1.11]	(0.04) [0.14, 0.30]	(0.39) [2.14, 3.69]	(0.39) [2.21, 3.75]	(0.15) [0.61, 1.18]	(0.35) [1.83, 3.20]	(0.34) [1.84, 3.19]	(0.13) [0.43, 0.94]
API Non-Hispanic	-0.37	-0.35	-0.35	-1.21	-1.18	-1.18	-1.09	-1.07	-1.06
	(0.08) [-0.53, -0.20]	(0.09) [-0.52, -0.18]	(0.03) [-0.41, -0.30]	(0.30) [-1.81, -0.62]	(0.30) [-1.77, -0.60]	(0.11) [-1.41, -0.96]	(0.28) [-1.63, -0.55]	(0.27) [-1.60, -0.54]	(0.10) [-1.26, -0.86]
Number of Observations	2230	2230	2230	2230	2230	2230	2230	2230	2230

NOTES: Authors' calculations, 2005-2019 WCIS and ACS. "API" = Asian/Pacific Islander. "TD/PD" = temporary disability/permanent disability. Heteroskedasticity-robust standard errors are in parentheses, and 95% confidence intervals are in brackets. Table reports predicted incidence rates and risk differences from Poisson regression models for lost-time injury counts. Estimates based on 15 multiply imputed datasets.

eTable 9: Sensitivity Analysis: Complete Records Analysis

Outcome	Lost-Time	Lost-Time	Lost-Time	Lost-Time	Lost-Time	Lost-Time
Exclude Unreliable Claim Administrators?	Y	Y	Y	Y	Y	Y
Exclude All Imputed Cases				Y	Y	Y
	Unadjusted?	Age/Sex-Adjusted?	Occupation-Adjusted	Unadjusted?	Age/Sex-Adjusted?	Occupation-adjusted
	Y			Y		
		Y	Y		Y	Y
			Y			Y
**Incidence Rates**						
White Non-Hispanic	0.82	0.82	0.82	0.74	0.74	0.74
	(0.06)	(0.06)	(0.02)	(0.06)	(0.06)	(0.02)
	[0.70, 0.94]	[0.70, 0.94]	[0.78, 0.86]	[0.63, 0.85]	[0.63, 0.85]	[0.71, 0.78]
Black Non-Hispanic	1.46	1.50	1.15	1.31	1.35	1.03
	(0.08)	(0.08)	(0.03)	(0.07)	(0.07)	(0.03)
	[1.30, 1.62]	[1.34, 1.66]	[1.09, 1.22]	[1.16, 1.45]	[1.20, 1.49]	[0.97, 1.08]
Hispanic	1.65	1.77	1.10	1.50	1.60	0.98
	(0.09)	(0.09)	(0.03)	(0.09)	(0.08)	(0.02)
	[1.48, 1.83]	[1.59, 1.94]	[1.04, 1.15]	[1.33, 1.67]	[1.44, 1.77]	[0.93, 1.02]
API Non-Hispanic	0.52	0.54	0.53	0.47	0.48	0.47
	(0.04)	(0.04)	(0.02)	(0.04)	(0.04)	(0.01)
	[0.44, 0.60]	[0.45, 0.62]	[0.50, 0.56]	[0.39, 0.54]	[0.40, 0.55]	[0.44, 0.50]
**Risk Difference Relative to White Non-Hispanic**						
Black Non-Hispanic	0.64	0.68	0.34	0.57	0.60	0.28
	(0.10)	(0.10)	(0.04)	(0.09)	(0.09)	(0.03)
	[0.45, 0.84]	[0.49, 0.88]	[0.26, 0.41]	[0.39, 0.75]	[0.42, 0.78]	[0.22, 0.35]
Hispanic	0.83	0.95	0.28	0.76	0.86	0.24
	(0.11)	(0.11)	(0.04)	(0.10)	(0.10)	(0.03)
	[0.62, 1.05]	[0.73, 1.16]	[0.21, 0.35]	[0.56, 0.96]	[0.66, 1.06]	[0.18, 0.30]
API Non-Hispanic	-0.30	-0.28	-0.29	-0.28	-0.26	-0.27
	(0.07)	(0.07)	(0.03)	(0.07)	(0.07)	(0.02)
	[-0.44, -0.15]	[-0.43, -0.14]	[-0.34, -0.24]	[-0.41, -0.14]	[-0.40, -0.13]	[-0.32, -0.23]
Number of Observations	2,230	2,230	2,230	2,230	2,230	2,230

NOTES: Authors' calculations, 2005-2019 WCIS and ACS. "API" = Asian/Pacific Islander. Heteroskedasticity-robust standard errors are in parentheses, and 95% confidence intervals are in brackets. Table reports predicted incidence rates and risk differences from Poisson regression models for lost-time injury counts. Estimates based on 15 multiply imputed datasets. Samples used in estimation restricted to cells with race/ethnicity-age-sex-occupation cells with FTE (over 2005-2019) above minimum thresholds reported in column headers. "Unreliable Claim Administrators" defined as those with paid benefits reported in under 15% of cases.

eTable 10: Sensitivity Analysis: Excluding Predominantly Public-Sector Occupations and Temporary/Contract Employees

Outcome	Lost-Time	Lost-Time	Lost-Time	Lost-Time	Lost-Time	Lost-Time
Exclude Public Sector Occupations	Y	Y	Y			
Exclude Temporary/Contract Workers				Y	Y	Y
	Unadjusted?	Age/Sex-Adjusted?	Occupation-Adjusted	Unadjusted?	Age/Sex-Adjusted?	Occupation-adjusted
	Y			Y		
		Y	Y		Y	Y
			Y			Y
**<u>Incidence Rates</u>**						
White Non-Hispanic	0.94	0.94	0.94	0.98	0.98	0.98
	(0.07)	(0.07)	(0.02)	(0.07)	(0.07)	(0.02)
	[0.81, 1.07]	[0.81, 1.07]	[0.89, 0.99]	[0.85, 1.12]	[0.84, 1.12]	[0.94, 1.03]
Black Non-Hispanic	1.71	1.75	1.35	1.69	1.73	1.34
	(0.10)	(0.10)	(0.04)	(0.09)	(0.09)	(0.04)
	[1.52, 1.90]	[1.56, 1.94]	[1.27, 1.42]	[1.52, 1.86]	[1.56, 1.91]	[1.26, 1.42]
Hispanic	1.89	2.03	1.24	1.82	1.96	1.26
	(0.10)	(0.10)	(0.03)	(0.09)	(0.09)	(0.03)
	[1.69, 2.09]	[1.83, 2.23]	[1.17, 1.30]	[1.65, 2.00]	[1.78, 2.13]	[1.19, 1.32]
API Non-Hispanic	0.60	0.62	0.59	0.62	0.63	0.63
	(0.05)	(0.05)	(0.02)	(0.05)	(0.05)	(0.02)
	[0.51, 0.70]	[0.52, 0.71]	[0.55, 0.62]	[0.52, 0.71]	[0.54, 0.73]	[0.59, 0.67]
**<u>Risk Difference Relative to White Non-Hispanic</u>**						
Black Non-Hispanic	0.77	0.81	0.41	0.70	0.75	0.35
	(0.12)	(0.12)	(0.05)	(0.11)	(0.11)	(0.05)
	[0.54, 1.00]	[0.58, 1.04]	[0.31, 0.50]	[0.48, 0.93]	[0.53, 0.97]	[0.26, 0.44]
Hispanic	0.95	1.09	0.30	0.84	0.97	0.27
	(0.12)	(0.12)	(0.04)	(0.11)	(0.12)	(0.04)
	[0.71, 1.19]	[0.85, 1.33]	[0.21, 0.38]	[0.62, 1.07]	[0.74, 1.20]	[0.19, 0.35]
API Non-Hispanic	-0.34	-0.32	-0.35	-0.37	-0.35	-0.35
	(0.08)	(0.08)	(0.03)	(0.09)	(0.09)	(0.03)
	[-0.50, -0.17]	[-0.48, -0.16]	[-0.41, -0.29]	[-0.54, -0.20]	[-0.52, -0.18]	[-0.41, -0.29]
Number of Observations	2,062	2,062	2,062	2,230	2,230	2,230

NOTES: Authors' calculations, 2005-2019 WCIS and ACS. "API" = Asian/Pacific Islander. Heteroskedasticity-robust standard errors are in parentheses, and 95% confidence intervals are in brackets. Table reports predicted incidence rates and risk differences from Poisson regression models for lost-time injury counts. Estimates based on 15 multiply imputed datasets. Samples used in estimation restricted to cells with race/ethnicity-age-sex-occupation cells with FTE (over 2005-2019) above minimum thresholds reported in column headers. " Public-Sector Occupations" are public safety and teaching occupations with greater than 50% employment in the public sector in the 2005-2019 ACS data used in our analysis (SOC codes 25-1000, 25-2000, 25-4000, 25-9000, 33-1000, 33-2000, and 33-3000). "Temporary/Contract" workers are those whose employer at injury is in NAICS industry 5613 (Employment Services).
