# Supplementary material for: Racial and Ethnic Disparities in Occupational Health
Source: JAMA Health Forum. 2025 Sep 26;6(9):e253495. doi: 10.1001/jamahealthforum.2025.3495 (PMC12475949; doi:10.1001/jamahealthforum.2025.3495)
Supplement: Supplement 3. — Data Sharing Statement [file jamahealthforum-e253495-s003.pdf]

Data Sharing Statement

Dworsky. Racial and Ethnic Disparities in Occupational Health. *JAMA Health Forum*. Published September 26, 2025. doi:10.1001/jamahealthforum.2025.3495

Data
Data available: No

Additional Information
Explanation for why data not available: The data used in this secondary study are highly sensitive and were accessed under a data use agreement with the State of California that prohibits us from redistributing the individual-level data. To help address this limitation in our ability to share data, we have submitted a spreadsheet as part of the supplement that contains aggregated estimates of injury rates and injury counts by occupation, gender, and race/ethnicity.
